# Longevity‐related molecular pathways are subject to midlife “switch” in humans

**DOI:** 10.1111/acel.12970

**Published:** 2019-06-06

**Authors:** James A. Timmons, Claude‐Henry Volmar, Hannah Crossland, Bethan E. Phillips, Sanjana Sood, Karolina J. Janczura, Timo Törmäkangas, Urho M. Kujala, William E. Kraus, Philip J. Atherton, Claes Wahlestedt

**Affiliations:** ^1^ Division of Genetics and Molecular Medicine King's College London London UK; ^2^ Scion House Stirling University Innovation Park Stirling UK; ^3^ Department of Psychiatry and Behavioral Sciences, Center for Therapeutic Innovation University of Miami Miller School of Medicine Miami Florida; ^4^ School of Medicine, Royal Derby Hospital University of Nottingham Derby UK; ^5^ Faculty of Sport and Health Sciences University of Jyväskylä Jyväskylä Finland; ^6^ Duke University School of Medicine Durham North Carolina

**Keywords:** aging, Alzheimer's, Brain, ECSIT, long noncoding RNA, mitochondrial complex 1, mTOR, reactive oxygen species, skeletal muscle, skin

## Abstract

Emerging evidence indicates that molecular aging may follow nonlinear or discontinuous trajectories. Whether this occurs in human neuromuscular tissue, particularly for the noncoding transcriptome, and independent of metabolic and aerobic capacities, is unknown. Applying our novel RNA method to quantify tissue coding and long noncoding RNA (lncRNA), we identified ~800 transcripts tracking with age up to ~60 years in human muscle and brain. In silico analysis demonstrated that this temporary linear “signature” was regulated by drugs, which reduce mortality or extend life span in model organisms, including 24 inhibitors of the IGF‐1/PI3K/mTOR pathway that mimicked, and 5 activators that opposed, the signature. We profiled Rapamycin in nondividing primary human myotubes (*n* = 32 HTA 2.0 arrays) and determined the transcript signature for reactive oxygen species in neurons, confirming that our age signature was largely regulated in the “pro‐longevity” direction. Quantitative network modeling demonstrated that age‐regulated ncRNA equaled the contribution of protein‐coding RNA within structures, but tended to have a lower heritability, implying lncRNA may better reflect environmental influences. Genes ECSIT, UNC13, and SKAP2 contributed to a network that did *not* respond to Rapamycin, and was associated with “neuron apoptotic processes” in protein–protein interaction analysis (FDR = 2.4%). ECSIT links inflammation with the continued age‐related downwards trajectory of mitochondrial complex I gene expression (FDR < 0.01%), implying that sustained inhibition of ECSIT may be maladaptive. The present observations link, for the first time, model organism longevity programs with the endogenous but temporary genome‐wide responses to aging in humans, revealing a pattern that may ultimately underpin personalized rates of health span.

## 1 INTRODUCTION

Aging is such an important “risk factor” for a number of chronic pathologies that enabling “healthy aging” represents a logical strategy to improve human health (Longo et al., 2015). In model organisms, regulators of longevity and health span have been extensively validated (De Haes et al., 2014; Schaar et al., 2015); these include inhibition of mTOR (Lamming, Ye, Sabatini, & Baur, 2013)—a nutrient and growth factor sensing, GTPase regulated protein complex (Pan & Finkel, 2017), which regulates “protective” autophagy programs (Yang et al., 2014), and strategies *down‐regulating* mitochondrial components accompanied by modest increases in reactive oxygen species (ROS) production (Arriola Apelo et al., 2016; Lamming et al., 2013). Interestingly, activation of the mTOR pathway has been reported in Alzheimer's disease (AD; Tramutola et al., 2015) and excessive TORC1 activity may contribute to muscle degeneration (Tang et al., 2019). In humans, age‐related molecular changes are typically modeled using linear methods, yet in shorter‐lived organisms (Hall et al., 2017; Manczak, Jung, Park, Partovi, & Reddy, 2005; Rana et al., 2017; Rangaraju et al., 2015; Yang & Hekimi, 2010) nonlinear molecular responses to age are observed (Rangaraju et al., 2015), featuring the aforementioned canonical pathways (Lamming et al., 2013; Pan & Finkel, 2017).

**Figure 1:**
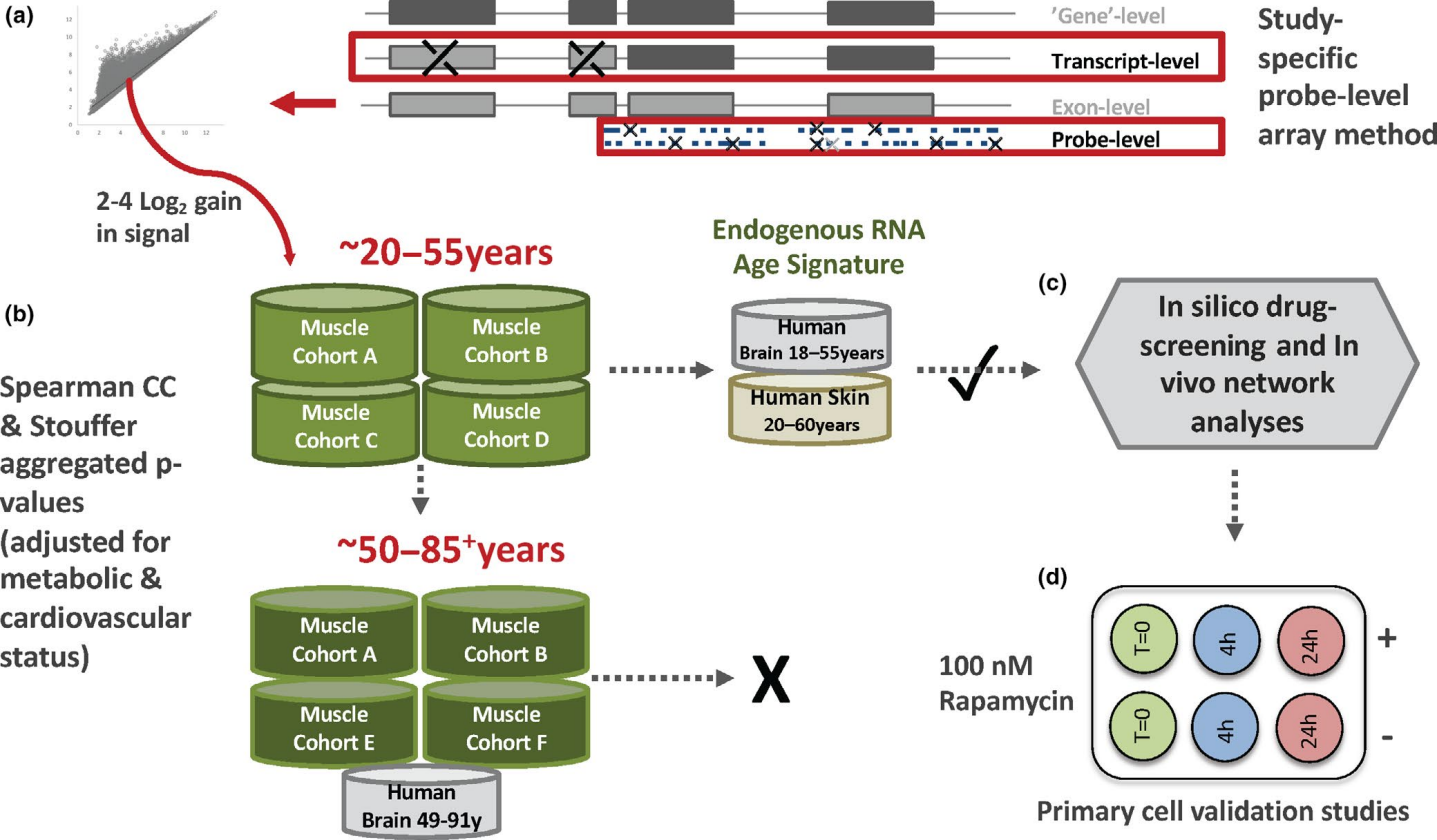
A schematic representation of the study analysis strategy. (a) For the HTA 2.0 or exon arrays, the 25‐mer array probes were realigned to the current genome; “single match” probes were GC content‐adjusted and study‐specific expression confirmed (low signal/variance filtering) *before* creating the template for combining probes into a transcript signal (selected from ensembl, ENST, Figure S1). (b) Linear modeling for “age” versus RNA was conducted using independent cohorts of human muscle profiles from physiologically characterized “healthy” drug‐free humans (*n* = 330 biopsies for decades third to sixth, *n* = 247 for decades sixth to ninth). The clinical data originate from our studies: Cohort A (Timmons et al., 2018), Cohort B (Phillips et al., 2013), Cohort C (AbouAssi et al., 2015), Cohort D (Phillips et al., 2017), Cohort E (Slentz et al., 2016), and Cohort F (Hangelbroek et al., 2016). The pattern of muscle age‐related transcript expression was confirmed in human brain (*n* = 299) and skin (*n* = 59), relying on published exon array data and our optimized transcript detection protocol. (c) An age‐related protein‐coding transcriptome was identified, adjusting for metabolic and aerobic capacity, and this provided a robust framework for characterization of the biology of age‐regulated lncRNAs, which are largely of unknown function, using network analysis and an age signature for in silico cMAP database drug screening. (d) The results of in silico drug screening were validated primary muscle cell studies

Beyond the need to consider different “phases” of molecular aging, clinical phenotypes such as aerobic capacity (Koch et al., 2011) and insulin resistance (Timmons et al., 2018)—highly variable environmentally sensitive and inherited traits—potentially interact with aging. Quantitatively important biomarkers for health, neither parameter has been previously available when modeling the molecular features of human aging. Furthermore, no study has utilized technology to both measure exon‐specific transcript signals and provide robust coverage of tissue long noncoding RNAs (lncRNAs, 50% of the human transcriptome; Timmons et al., 2018; Deveson, Hardwick, Mercer, & Mattick, 2017). Furthermore, emerging evidence demonstrates that lncRNAs can modulate mTOR activity (Chen et al., 2018; Li et al., 2016). These factors could combine to explain why existing models of human aging do not consistently identify a molecular program dominated by the canonical regulators of longevity in nonhuman systems. In the present study, we combine our advanced RNA methodology (Figure 1a) with the production of physiological data at scale, to model these three interacting phenotypes (Figure 1b). This revealed a molecular program in three human tissue types *dominated* by mTOR and ROS signaling, including selective loss of mitochondrial complex I gene expression.

## 2 RESULTS

### 2.1 A linear protein‐coding RNA response to aging is switched off by the sixth decade of life

**Figure 2:**
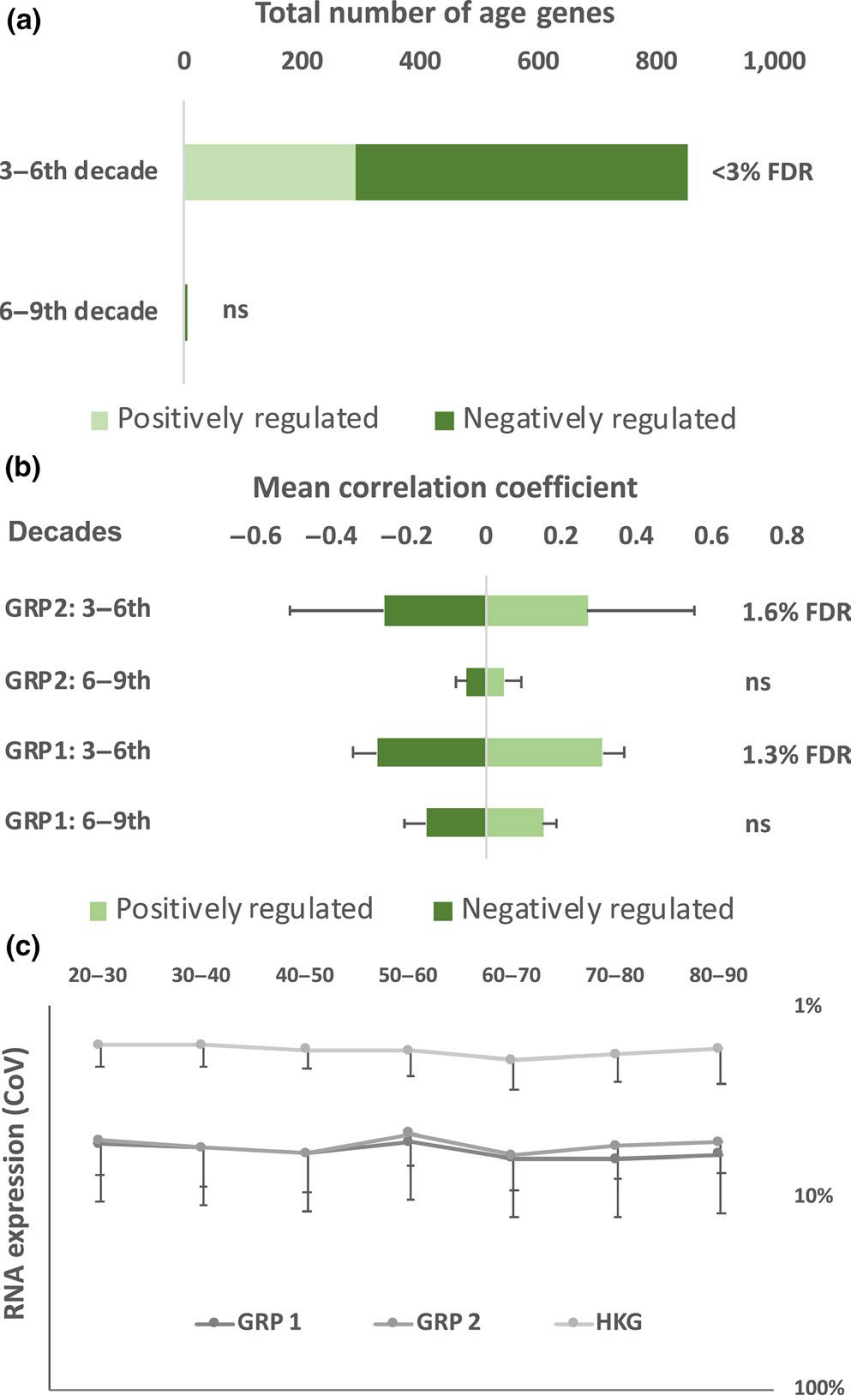
Linear modeling and protein‐coding transcript expression variation in 577 adult human muscle RNA samples. (a) Linear modeling applied over two‐ three‐decade periods of adulthood identified a statistically significant gene expression program during the first period but not in the second. (b) Two‐thirds of the Spearman rank correlation coefficients were negatively associated with age during the third to sixth decade of adulthood (FDR < 3%). A minority of these were related in a similar, numerical, manner to age, from the sixth decade (“Group 1 genes”), while the majority (557 from 853) had zero correlation with age beyond the sixth decade (“Group 2 genes”). HKG were potential neuromuscular “housekeeping genes” identified as demonstrating a very low coefficient of variation across age, in both tissues. (c) The coefficient of variation for the transcript expression values of the age transcripts were calculated for the two age‐regulated gene sets and found to be similar and stable across seven decades of adulthood. This is evidence that the observed age‐related pattern was not due to transcriptional stochasticity (“noise”) but due to the active switching‐off of a transcriptional program regulating Group 2 genes

We first examined the *protein‐coding* transcript responses during the first and second 30‐year time spans of adulthood (20–55 years, *n* = 330, Figure 1b), a choice ensuring a similarly large sample for analyzing the following 30‐year period. The RNA‐versus‐age relationship was adjusted for insulin sensitivity and aerobic capacity (Phillips et al., 2017). This identified 1,967 ENSTs consistently age‐related across four clinical cohorts (Figure 2a, mean FDR 1.3%, Appendix S1), representing 694 protein‐coding genes, of which two‐thirds *declined* over three decades. This adjusted “linear” age‐related signature included components of the mTORC1 pathway (LAMTOR5/HBXIP)—a regulator of protein translation and cellular autophagy (Zoncu, Efeyan, & Sabatini, 2011)—and members of the mTORC2 pathway (MAPKAP1; mSIN1)—a regulator of apoptosis and substrate metabolism (Liu, Gan, et al., 2013). Background bias‐adjusted ontology analysis (Timmons, Szkop, & Gallagher, 2015) identified down‐regulated mitochondrial complex I (12.8 times enrichment, FDR < 0.01%) and mitochondrial translation (9.9 times enrichment, FDR < 0.01%) processes. Using the only human brain dataset with this age‐range *and* exon‐based transcript data (Kang et al., 2011), we examined these 1,967 ENSTs in cerebellum, hippocampus, and frontal cortex (18–55 years; *n* = 116; Appendix S2). Despite the more limited sample size, 47% of the age genes were regulated in an identical manner to our observations in muscle (Appendix S3). Skin, like brain, is of ectodermal origin and re‐modeling of an exon array dataset (Haustead et al., 2016) found that 57% of the age genes were regulated in a manner consistent with muscle aging (*n* = 59, drug‐free subjects, Appendix S2). Thus, a linear protein‐coding gene expression program, containing model organism longevity genes, is identifiable in human tissue aging during the first three decades of adulthood (Figure 2a).

Applying the same analysis approach across the subsequent three decades (51–86 years, *n* = 247), it was observed that *none* of the 73,654 protein‐coding ENSTs demonstrated a statistically significant relationship with age in skeletal muscle (the lowest FDR was 9%, Figure 2a). Undiagnosed disease could result in stochastic gene expression (reducing statistical power), so we modeled only the 1,967 ENSTs. Five now reached a modest level of statistical significance: MLF1, HEXIM2, TMEM266, MYLK4, and GRSF1 (<10%FDR). Critically, on laborious visual inspection, a majority (76%) of the 1,967 transcripts (507 genes) demonstrated close to a zero correlation coefficient with age beyond the sixth decade (referred to as “Group 2” genes, Appendix S1, Figure 2b), while 24% had similar trajectories over both periods (“Group 1” transcripts, Figure 2b). The coefficient of variation for RNA expression (Figure 2c) for Group 1 did not differ from the Group 2, implying specific termination of interaction with age for Group 2 genes. For human brain, the only sufficiently sized older age‐range exon‐based dataset was from Hardy and colleagues (Trabzuni et al., 2011). One hundred and eighty‐three samples from the same three brain regions used above, passed quality checks (cerebellum, hippocampus, and frontal cortex; 49–91 years, Appendix S4). Fifty‐two Group 1 age genes were consistently regulated in brain and muscle (36% of those detected), while 190 Group 2 age genes (64% of those detected) had a consistent relationship with age, in muscle and brain (Appendix S4). Thus, a linear gene expression program active during the first three decades of adulthood is largely “switched off” in human neuromuscular tissue, from the sixth decade of life.

### 2.2 In vitro and in silico analyses demonstrate that the human age signature is regulated by proven mediators of model organism longevity

Multiple independent resources were utilized to provide insight into the regulators of this human age signature. Reactive oxygen species (ROS) are generated in mitochondrial respiratory chain Complex I, and down‐regulated Complex I genes were a highly enriched component of Group 2 genes (14.8× enriched, *p* < 1 × 10^−9^). Paraquat increases superoxide production in vitro (Lenzken et al., 2011), and updated analysis of data from neuronal cells treated for 18 hr found ~60% of the expressed protein‐coding age transcripts (*n* = 461) were regulated by ROS (vs. ~20% of all transcripts, Appendix S3, FDR ≤ 1%). Strikingly, 19 mitochondrial complex I genes were regulated by Paraquat in the same direction as age, in vivo. Upstream analysis, used as previously described (Nakhuda et al., 2016), identified RICTOR activation (*p* < 1 × 10^−11^, Z‐score = 4.03)—a component of mTORC2 required for the function of long‐term memory (Huang et al., 2013)—and two synthetic retinoids, ST1926 (*p* < 1 × 10^−4^, Z‐score = 3.0) and CD437 (*p* < 1 × 10^−3^, Z‐score = 3.0). In contrast, XBP1 was predicted to be upstream but inhibited (*p* < 1 × 10^−4^, Z‐score = −2.38); XBP1 is a transcriptional component of the unfolded protein response (Rana et al., 2017).

**Figure 3:**
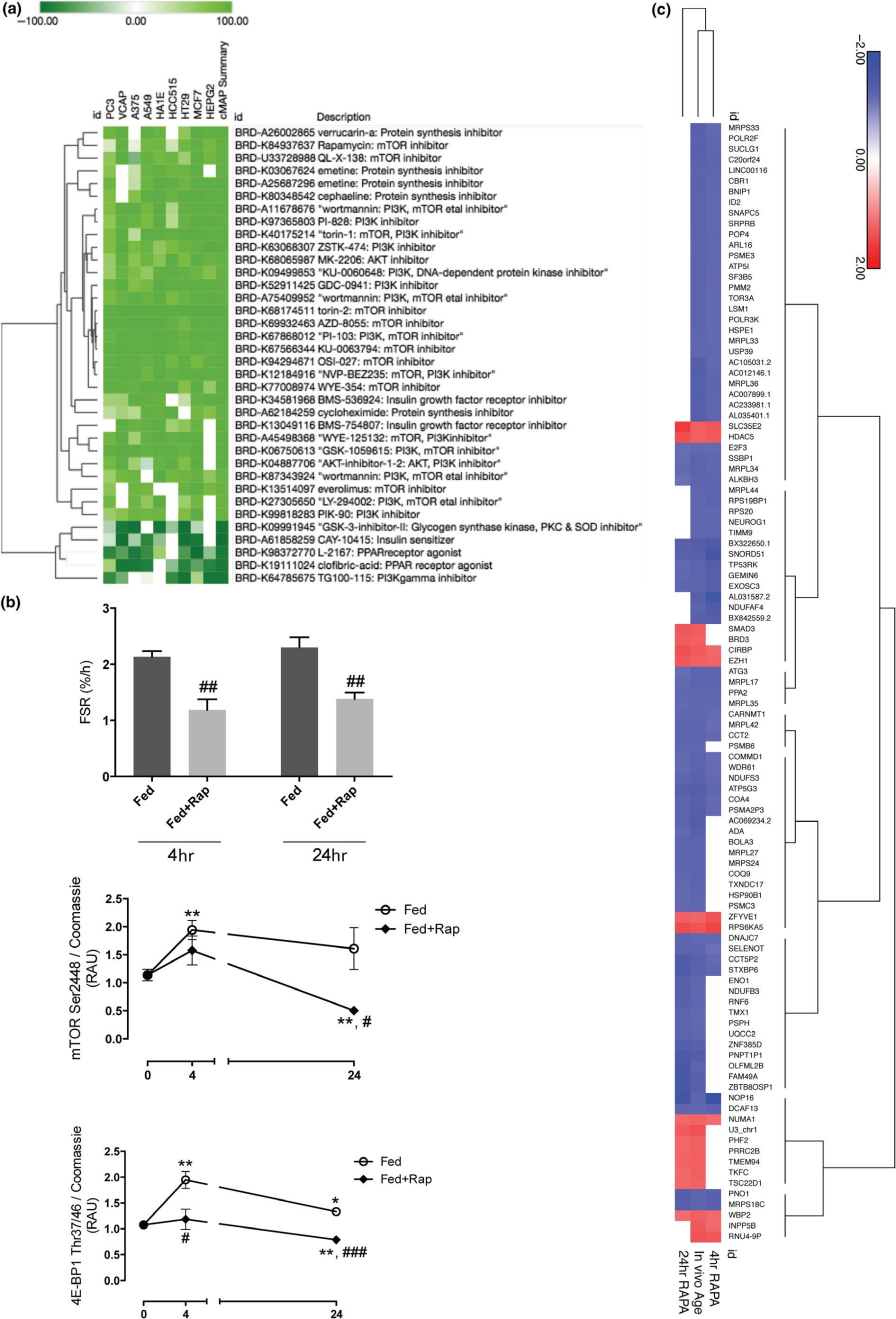
Discovery and validation that the in vivo muscle age signature is largely regulated by the canonical IGF1/PI3K/mTOR “longevity‐related” pathway. (a) The protein‐coding age transcripts were used as a signature to match to the CMap‐L1000v1 database of >8,000 chemicals profiled in nine distinct cell lines. The maximum possible scores are −100/100 and only 169 drugs (<2%) scored above −90/90. The linear age signature matched proven longevity canonical signaling pathway (IGF1–PI3K–mTOR); 24 “inhibitory” compounds mimicked the first period of aging, and five compounds activating aspects of the IGF1–PI3K–mTOR pathway opposed the in vivo pattern. (b) The relationship between the activity of mTOR pathway and the age transcripts was evaluated in human primary muscle myotubes, using rapamycin (100 nM). Relative changes in phosphorylation of mTOR Ser2448 and 4E‐BP1 Thr37/46 following IFG1/amino acid feeding, confirming the activity of rapamycin (RAU = relative arbitrary units; for 4E‐BP1). Protein data represent experiments using four independent experiments per treatment and time‐point. *p < 0.05, **p < 0.01, ***p < 0.001 versus baseline, respectively. #*p* < 0.05, ##*p* < 0.01, ###*p* < 0.001 versus time‐matched control group, respectively. (c) RNA was isolated from eight independent experiments (per treatment/time‐point) and profiled on the HTA 2.0 array (*n* = 40 arrays). Overlap between primary muscle rapamycin‐regulated transcripts (up/down‐regulation) and the in vivo age signature (positive/negative correlation) was evaluated at 4 and 24 hr. For Group 2 age transcripts, the pattern of expression after 4 hr rapamycin treatment clustered more closely with the in vivo age signature (more likely only mTORC1), while the Group 1 genes clustered with the 24‐hr in vitro signature, when activity mTORC2 can also be affected via depletion of TOR kinase

We used Group 1 and Group 2 age signatures in CMap‐L1000v1 (https://clue.io/) to establish whether they matched the RNA signatures for >8,000 cell line drug‐screening assays (Corsello et al., 2017). This analysis identified 24 inhibitors of the IGF‐1/PI3K/mTOR longevity‐regulating pathway across the nine cell lines, a striking observation as only 55 compounds in CMap‐L1000v1 are listed to inhibit this pathway (Appendix S5). The 24 inhibitors included rapamycin, an mTORC1 inhibitor, and Torin2, a direct active site inhibitor of mTOR kinase (Liu, Xu, et al., 2013). In addition, five compounds which *activate* IGF‐1/PI3K/mTOR pathway components, *opposed* our age signature (Figure 3a), confirming the bi‐directional relationship between pathway status and our in vivo signature.

To validate these in silico results from cell lines in terminally differentiated cells, we studied mTOR inhibition in human primary postmitotic myotubes (*n* = 32). Treated with IGF1 and amino acids, with or without 100 nM rapamycin (4 hr and 24 hr, Figure 3b), the coding and lncRNA transcriptome was profiled using the same technology as the clinical studies (*n* = 32). We observed that 106 Group 2 age genes (46% of the Group 2 genes expressed in vitro) and 21 of the 83 Group 1 genes were responsive to rapamycin. Hierarchical clustering (Figure 3c) indicated that Group 2 age transcript responses more closely resembled short‐term rapamycin treatment (4 hr, Figure 3c), while Group 1 age transcript responses were more closely associated with a 24‐hr rapamycin exposure (Figure S2). In contrast, when a large and robust human muscle insulin resistance RNA signature (Timmons et al., 2018) was utilized as a control input for tissue‐related bias (Timmons et al., 2015), very few compounds were significant (Appendix S5).

### 2.3 Network and heritability analysis reveals potential functions for noncoding RNA

**Figure 4:**
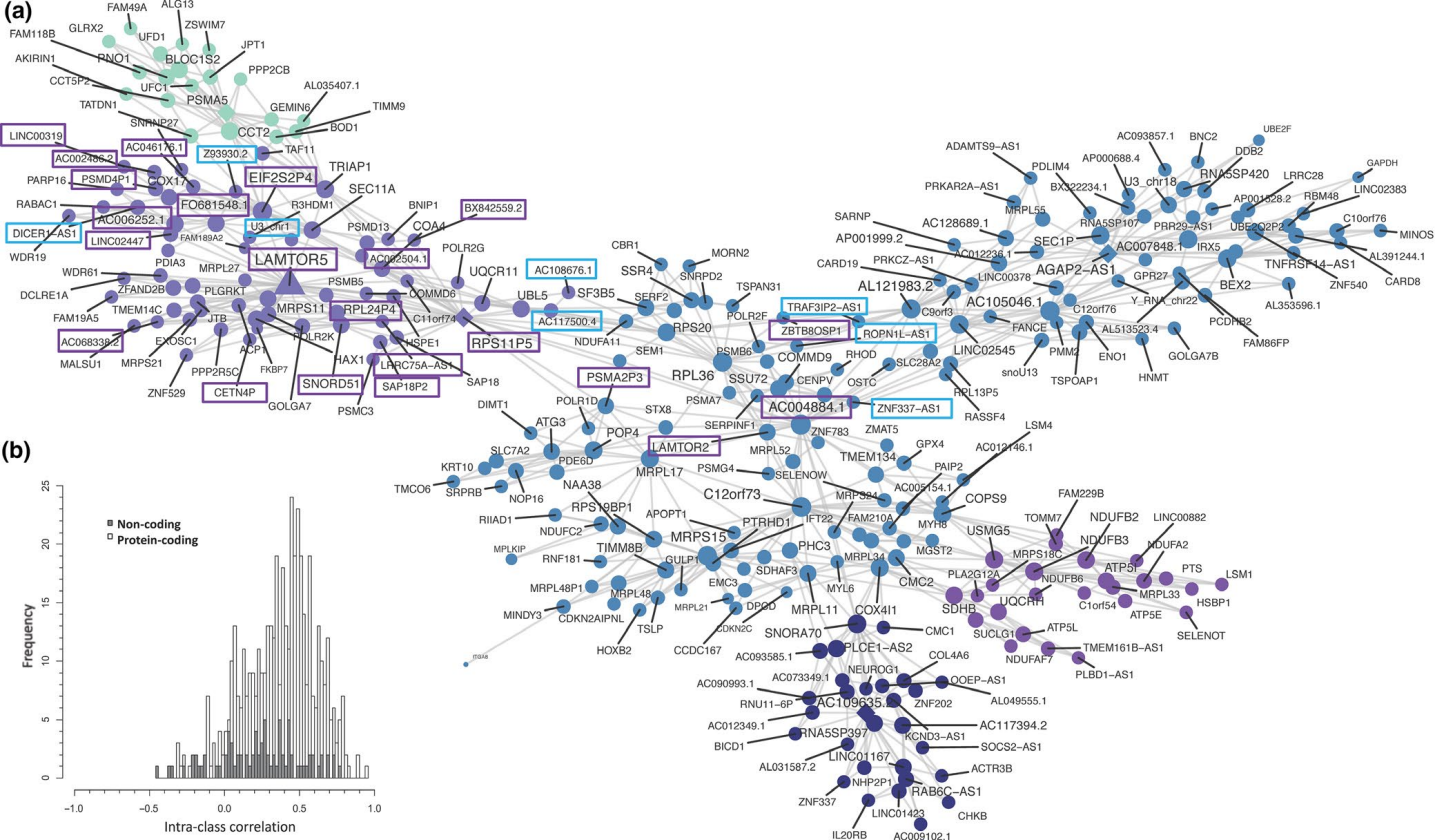
LAMTOR5 and associated lncRNAs. (a) Network structures within the coding/lncRNA transcript expression data were evaluated (*n* = 185, <68 years, FDR < 1% for Spearman correlation; *p* < 0.01 for module significance and *p* < 0.01 for network connectivity and 10,000 permutations for calculating FDR and connectivity p‐values) and plotted using a Fruchterman–Reingold force‐directed method (Song & Zhang, 2015). A network was identified, containing components of the mTOR pathway (LAMTOR5 and LAMTOR2, which decline with age). LAMTOR5 was strongly co‐regulated with >22 lncRNA (blue rectangle positively regulated with age; purple rectangle genes decline with age), including antisense, lincRNA, and RNA pseudogenes related to the translational machinery. Additional modules included mitochondrial, endoplasmic reticulum, and ribonuclear proteins with ncRNA of unknown function closely integrated with these protein‐coding genes. (b) Intraclass correlations (ICCs) of age‐regulated genes as indicators of heritability plotted for noncoding and protein‐coding expression using HTA 2.0 blood RNA profiles obtained from 17 pairs of homozygotic twins (Sood et al., 2016). The heritability estimate was greater for protein‐coding genes (*p* < 0.0001, See Table S2)

A subset of samples (*n* = 238, Figure 1 and Table S1) was profiled on the latest generation technology, enabling the study of genome‐wide lncRNA relationships with age. Our RNA quantification method detects ~15,000 ncRNAs across brain and muscle (Figures S4 and S5), five times more than short‐read RNA‐seq (Deveson et al., 2017; Jaffe et al., 2014). After accounting for variations in aerobic and metabolic fitness in subjects aged 18–51 years (*n* = 124, Table S1), 239 ncRNA transcripts (180 noncoding genes) were age‐related; this included 43 natural antisenses and 36 long intergenic RNAs (Appendix S6). The relationship with age for these ncRNAs was examined in older subjects (Cohort E (*n* = 68, 45–75 years) and Cohort F (*n* = 46, 65–86 years), Table S1). Again, many of ncRNAs no longer linearly correlated with age later in life, while interestingly 71 ncRNA transcripts demonstrated a Group 1 type profile. LncRNA responses were integrated with protein‐coding aging transcripts using quantitative network analysis (Song & Zhang, 2015). We used the largest possible batch of samples (18–67 years, *n* = 185, median age = 43 years), modeling the 840 coding and noncoding age transcripts, and discovered that the node statistics for lncRNA genes equaled those of the protein‐coding genes (as well as subsets such as “mitochondrial genes” and “in vitro rapamycin‐responsive” genes, Appendix S7). Thus, lncRNAs equally contribute to the network structure of the muscle age regulated transcriptome. Numerous lncRNAs were quantitatively co‐regulated with components of the mTOR canonical and protein synthesis pathways (Figure 4). For example, the mTOR amino acid sensing Ragulator complex gene, LAMTOR5 (Li et al., 2016), was down‐regulated from the third to sixth decade and densely associated with lncRNAs (*n* = 22). Using blood gene expression data from monozygotic twins (Sood et al., 2016), we conducted pilot heritability analyses (Figure 4b, Table S2, and Figure S6). Heritability of the age‐related gene expression—estimated from intraclass correlation analysis using blood RNA—was less for lncRNAs (*p* = 2.2 × 10^−16^, mean difference: −0.2756 [95% CI: −0.2828, −0.2685]), compared with age‐related protein‐coding gene expression. This indicates that altered regulation of lncRNAs may better reflect environmental than genetic influences during human aging.

## 3 DISCUSSION

We find that humans endogenously activate a transcriptional program related to enhanced longevity in model organisms and this “switches off” in human muscle and brain around the sixth decade of life. There is support for functionally important age “switches” in *Drosophila,* where selective midlife induction of mitochondrial fission via over‐expression of the GTPase gene, drp1 (DNM1L in humans), extends lifespan (Rana et al., 2017). Modulation of GTP and ROS can subsequently impact on aging via mTOR activity and mitochondrial redox signaling (Wang, Yang, & Zhang, 2016). Our modeling approach was pragmatic, relying on two large groups of samples to examine the “early” and “later” phase of human neuromuscular aging. Our signature identified drugs used to treat age‐associated diseases (Figure S3b, Appendix S5), for example, nitrendipine (Tuomilehto et al., 1999), losartan (Lindholm et al., 2002), and fluvastatin (Arampatzis et al., 2005)—all of which reduce mortality. Aliper *et al* assayed for potential anti‐age compounds using an artificial intelligence (AI) approach (Aliper et al., 2016), finding HA‐1004 (calcium channel blocker) and Fasudil (HA‐1077), both of which regulate our Group 2 age genes. Using a similar approach to search for natural mimetics of rapamycin they identified Withaferin A (Aliper et al., 2017), and Withaferin A was our top‐ranked hit, while another 11 other drugs were common to both projects. Together, these observations demonstrate that chemicals, which extend longevity (Arriola Apelo et al., 2016; Lamming et al., 2013; Lesniewski et al., 2017; Majumder et al., 2012) or drugs that reduce mortality in human clinical trials, also regulate our human age‐related transcriptional signature, suggesting it could be an endogenous pro‐survival program.

The mitochondrial and Toll pathway protein ECSIT has been hypothesized to be a disease hub in dementia (Soler‐López, Badiola, Zanzoni, & Aloy, 2012) because it reflects a point of interaction for inflammation and mitochondrial biology. ECSIT (down‐regulated with age) was the *top‐ranked* hub gene in the age transcriptome (Figure S7 and Appendix S7). Composed of 209 genes, the ECSIT network included CADM2, UNC13C and ST3GAL3 genes, with variants linked to cognition (Pasanen et al., 2018). ECSIT promotes NFκB activity (Wi et al., 2014), and in AD experimental models, repression of NFκB activity decreases BACE1 activity and both soluble and insoluble Aβ (Paris et al., 2010). Loss of ECSIT tempers mitochondrial Complex I assembly (Vogel et al., 2007), and modulation of Complex I results in changes in mitochondrial ROS production (Yang & Hekimi, 2010). Reactive oxygen species links mitochondrial function and the unfolded protein response (uPR) with aging and AD (Kennedy & Lamming, 2016; Miwa et al., 2016), and excess ROS generated in mitochondrial respiratory chain complex I (Kennedy & Lamming, 2016; Miwa et al., 2016) can cause neuronal death. However, moderate increases in mitochondrial ROS induce pro‐longevity pathways (Heidler, Hartwig, Daniel, & Wenzel, 2010; Schaar et al., 2015; Yang & Hekimi, 2010).

Chronic inhibition of ECSIT, perhaps due to excess "inflammation", may ultimately compromise Complex I function (Geng et al., 2015; Soler‐López et al., 2012; Wi et al., 2014). Earlier non‐linear‐based approaches identified a 150‐gene protein‐coding aging signature (Sood et al., 2015) including >30 genes subsequently linked to aging or dementia [See online supplement for citations]. As expected, only a few of these genes are present in our linear “*age‐switch”* model (UNC13C, MAPKAP1, SIN3A, PRKAR2A, MAPRE3, PCDH9, MSI2, and SKAP2). UNC13C and SKAP2 are particularly interesting as both are regulated by exercise training (unlike the majority of Group 1 or 2 age genes, Figure S8 and Appendix S8); however, ECSIT–UNC13C–SKAP2 represent a core of Group 1 age genes that *do not* respond to Rapamycin treatment in vitro*,* while protein–protein interaction analysis (Xia, Benner, & Hancock, 2014) indicates they can be associated with “neuron apoptotic processes” (Figure S9, FDR = 2.4%, Appendix S9).

Our RNA data‐processing approach produces a more comprehensive map of the lncRNA transcriptome than short‐read RNA‐seq approaches (FigureS 1, S4 and S5). Numerous lncRNAs were quantitatively co‐regulated with mTOR‐related genes, included pseudogenes of the protein translation machinery (Figure 4a) which act as decoys for miRNAs and RNA binding proteins (Zheng et al., 2018). Five lncRNA neighbors of LAMTOR5 were down‐regulated with age *and* rapamycin treatment (EIF2S2P4, SNORD51, FO681548.1, AC046176.1, and BX842559.2, Figure 4a and Appendix S7), while AC068338.2 and the U3 snoRNA (from chromosome 1) were upregulated by rapamycin. U3 is upregulated with age until the sixth decade of life and is a regulator of 18 s rRNA folding during ribosome biogenesis (Dutca, Gallagher, & Baserga, 2011). In contrast, LINC00319 is down‐regulated with age and promotes tumor growth via transcriptional silencing (Zhang et al., 2018). Given the emerging evidence that lncRNAs help direct mTOR specificity in vitro (Chen et al., 2018; Li et al., 2016), this suggests that our age‐regulated lncRNAs can fine‐tune the regulation of longevity‐related proteins.

In conclusion, we identify a molecular signature active up to the sixth decade of human life that largely dissipates thereafter. Representing inhibition of mTOR (and other strategies), excessive loss of activity might be predicted to impair metabolic homeostasis through, among other things, depletion of skeletal mass in gravity‐sensitive humans. Whether this juxtaposition underpins the midlife switch‐off that we have observed remains to be determined. Regulating this age signature perhaps through a combination of already existing drugs may provide an achievable and cost‐effective means of promoting healthy aging and delaying dementia. On the other hand, the natural termination of the signature, by midlife, may indicate that it has outlived its usefulness.

## 4 EXPERIMENTAL PROCEDURES

Extended data analysis methods are provided online and utilized numerous informatics resources (Bengtsson, Simpson, Bullard, & Hansen, 2008; Dai et al., 2005; Gentleman et al., 2004; Wang et al., 2012). All clinical studies complied with the 2008 Declaration of Helsinki, and RNA profiling was approved by the relevant ethics committees stated in each clinical article; all participants provided written informed consent (AbouAssi et al., 2015; Phillips et al., 2017, 2013; Slentz et al., 2016; Timmons et al., 2018). An overview of the analytic steps can be found in Figure 1, and the clinical characteristics can be found in Table S1. The HTA 2.0 array data have been deposited at GEO (GSE104235 and GSE130789) including (*n* = 32, plus 8 nontreated controls, GSE130789) the primary skeletal muscle cell rapamycin study. Our existing array data are available at GEO (GSE47969, GSE47881, GSE48278, GSE18732, GSE73142). We utilized two human brain public domain datasets on exon arrays from GEO (GSE25219 and GSE46706): one neuronal cell line data on HTA 2.0 (GSE21450) and one human skin dataset (E‐GEOD‐18876), also on exon arrays. Our muscle cell studies were carried out as previously reported (Crossland, Timmons, & Atherton, 2017). We conducted the CMap analysis using a database of ~8,000 chemical perturbagens (CMap‐L1000v1) to identify chemical compound mediators that mimic or oppose the linear age protein‐coding signature (https://clue.io/; Corsello et al., 2017). Our recently published human insulin resistance RNA signature (Timmons et al., 2018) was used as a comparator to control for tissue‐related gene expression bias (Timmons et al., 2015). We used the R‐package MEGENA (Song & Zhang, 2015) to identify network structures (FDR < 1% for Spearman correlation; *p* < 0.01 for module significance, and *p* < 0.01 for network connectivity and 10,000 permutations for calculating FDR and connectivity p‐values), and network data plots were produced using Fruchterman–Reingold force‐directed plotting.

## CONFLICT OF INTEREST

None declared.

## AUTHOR CONTRIBUTIONS

The human muscle transcriptomic biobank project was initiated by JAT, WEK, PJA, and CW (1997 onwards). Data processing and clinical data were collated, quality checked, and modeled by JAT. JAT, HC, and PJA were responsible for planning the cell biology work, and HC carried out the work. The primary statistical analysis was carried out by JAT. TT and UMK were responsible for twin data collection and analysis. The secondary data analysis and literature assessments were carried out by JAT, CW, KJJ, PJA, and CHV, while the manuscript was drafted by JAT, CHV, PJA, WEK, and CW, and edited by all authors, who contributed to reading, editing, and approval of the final version of the manuscript.
