# Supplementary figures and images for: Longevity‐related molecular pathways are subject to midlife “switch” in humans
Source: Aging Cell. 2019 Jun 6;18(4):e12970. doi: 10.1111/acel.12970 (PMC6612641; doi:10.1111/acel.12970)

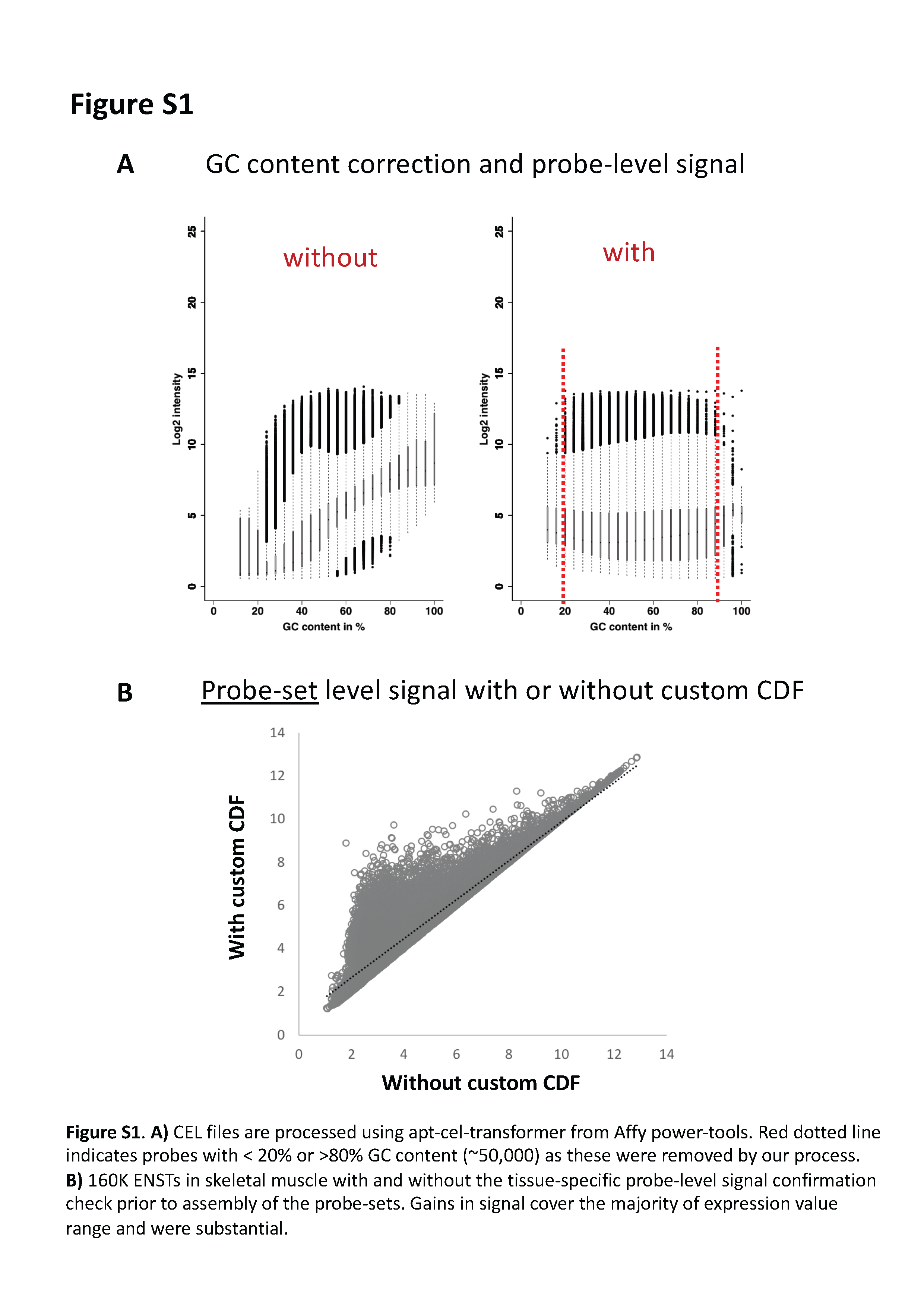

Supplement: Supplementary file 1 [file ACEL-18-e12970-s001.tiff]

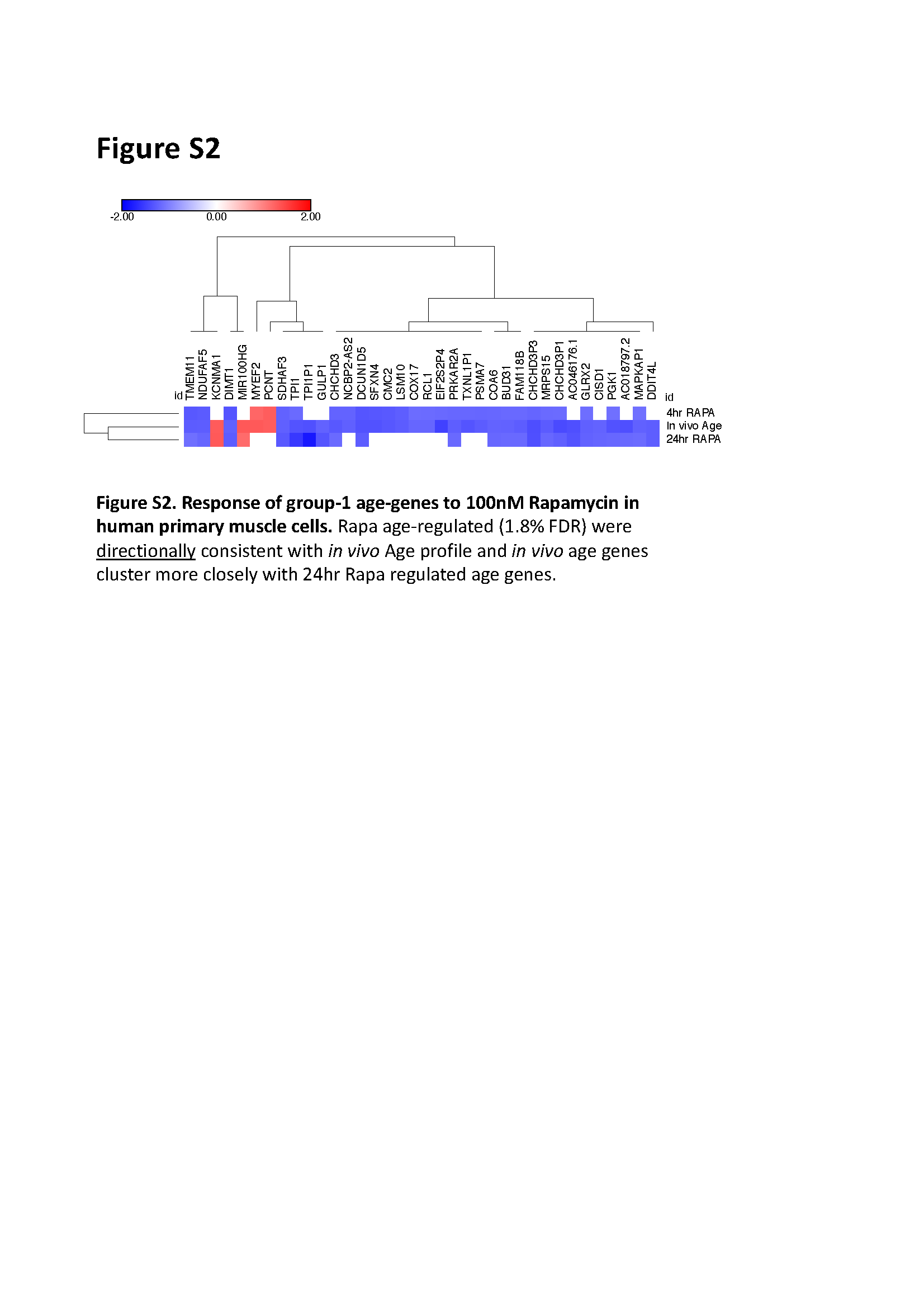

Supplement: Supplementary file 2 [file ACEL-18-e12970-s002.tiff]

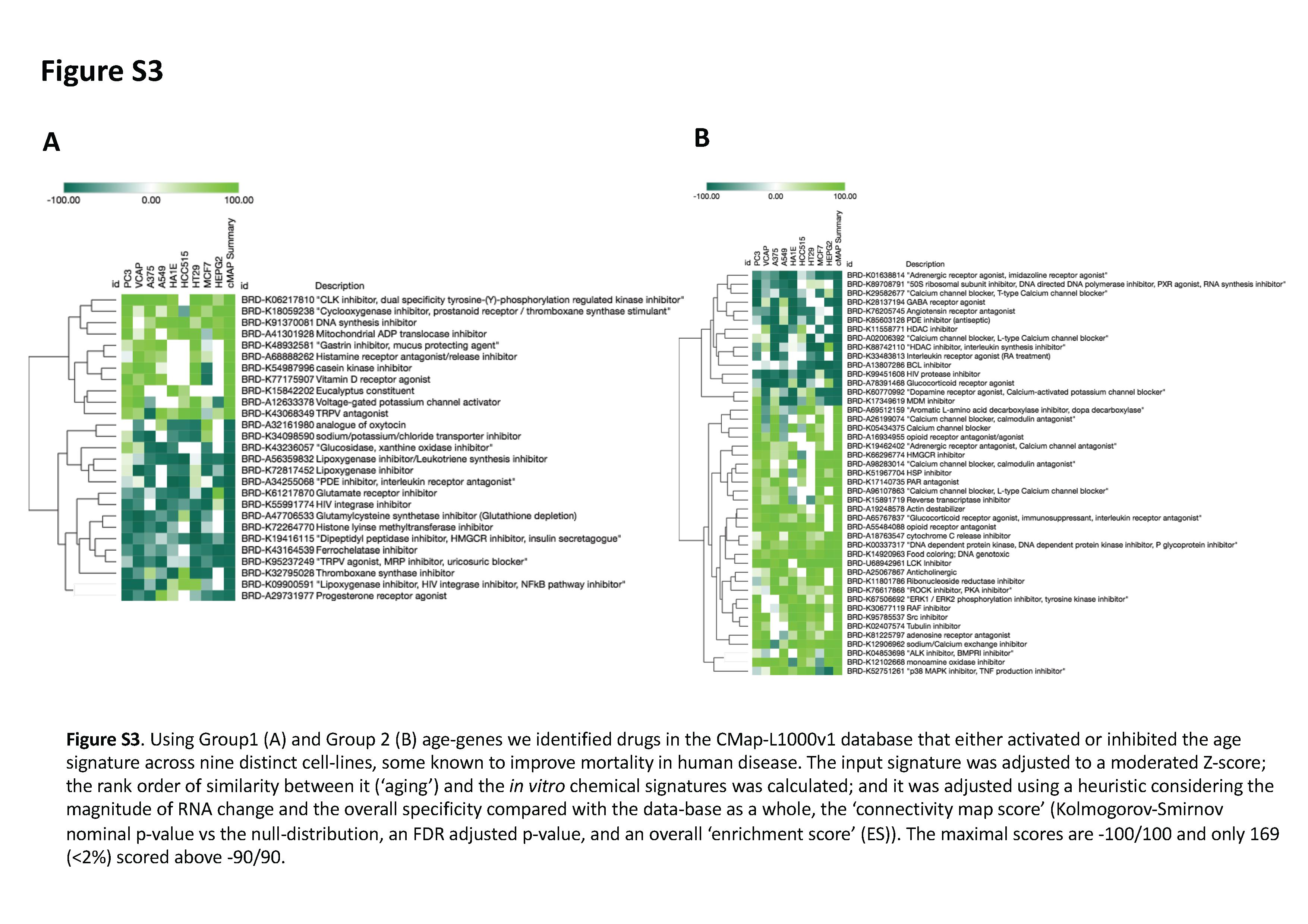

Supplement: Supplementary file 3 [file ACEL-18-e12970-s003.tiff]

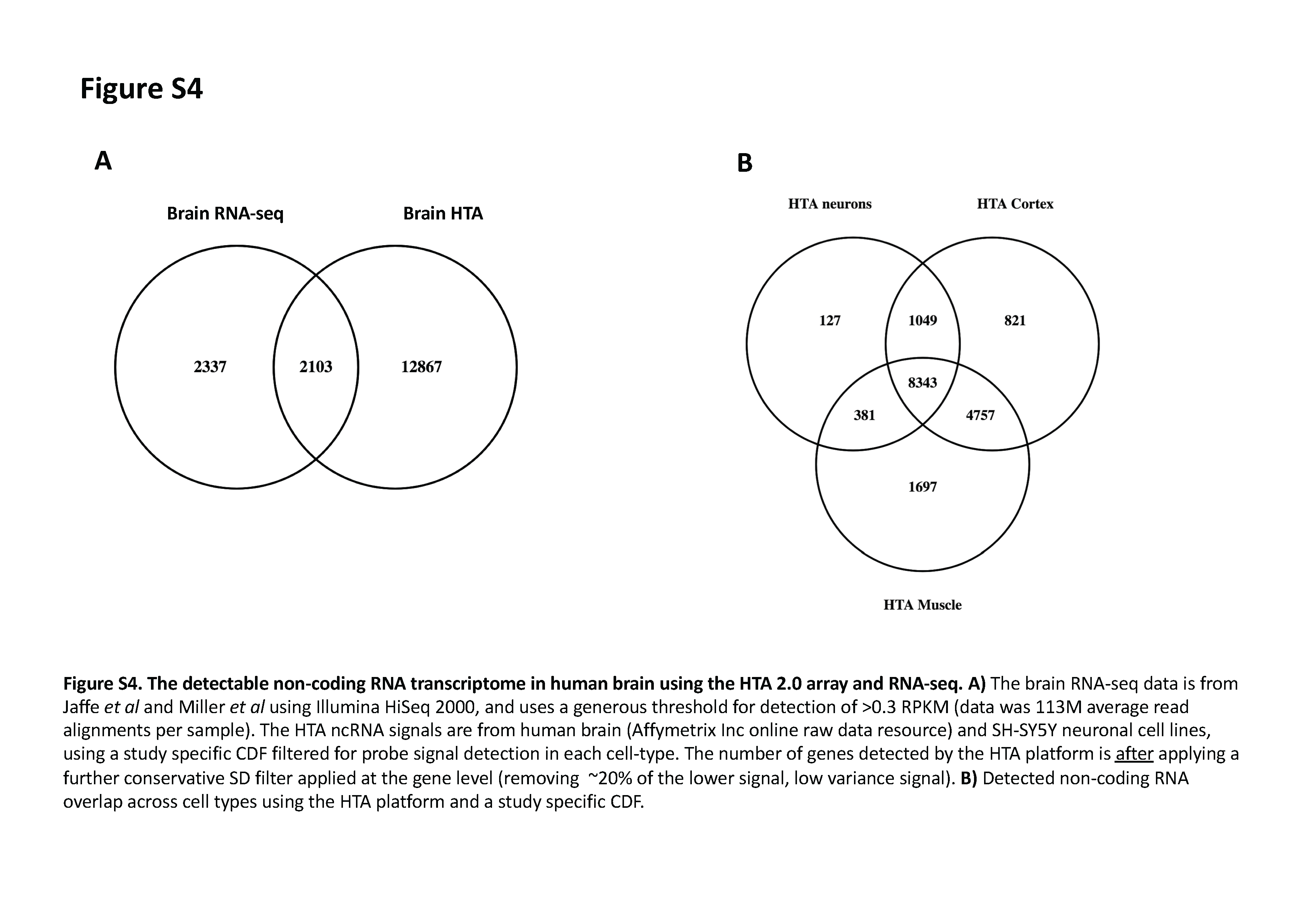

Supplement: Supplementary file 4 [file ACEL-18-e12970-s004.tiff]

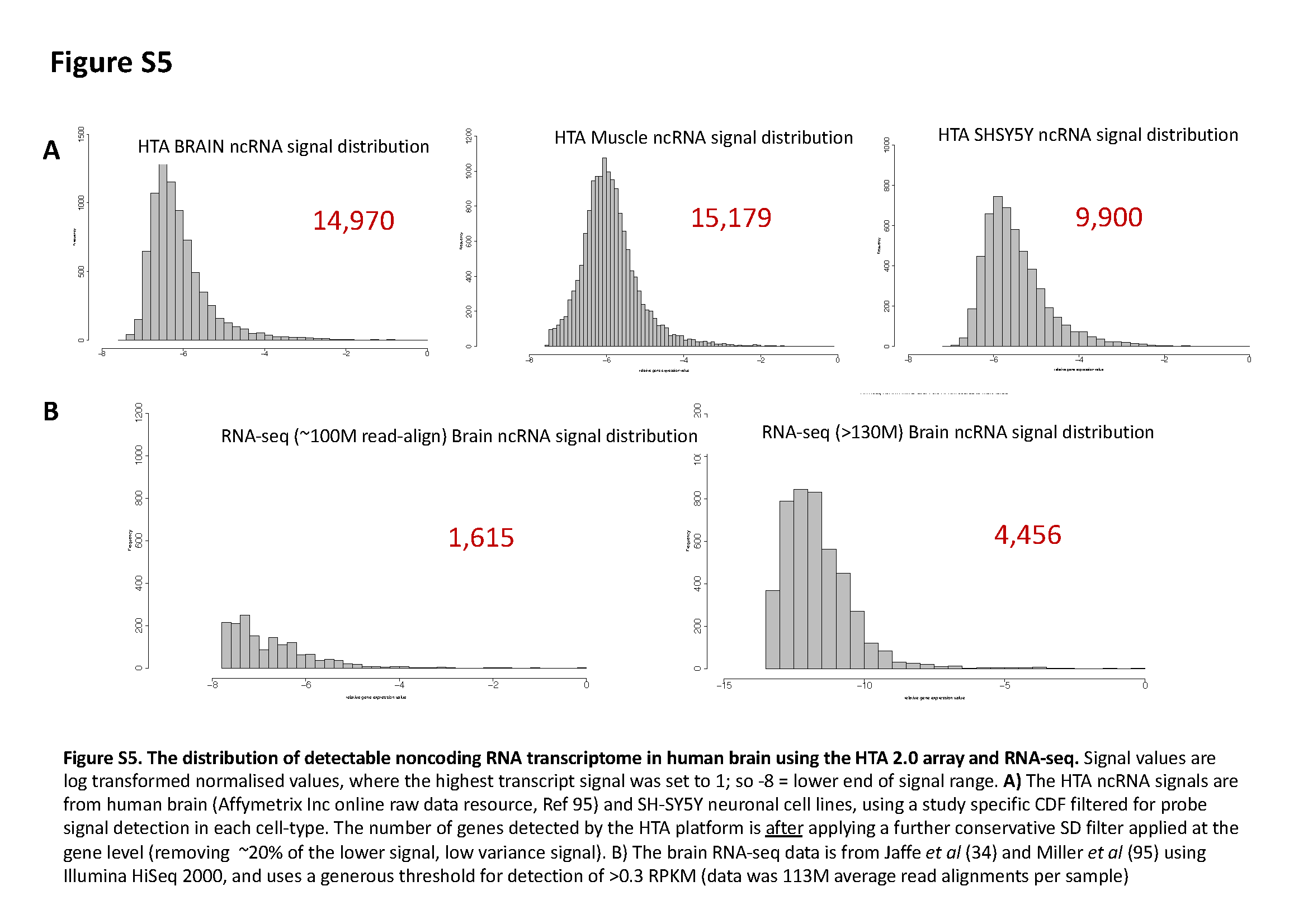

Supplement: Supplementary file 5 [file ACEL-18-e12970-s005.tiff]

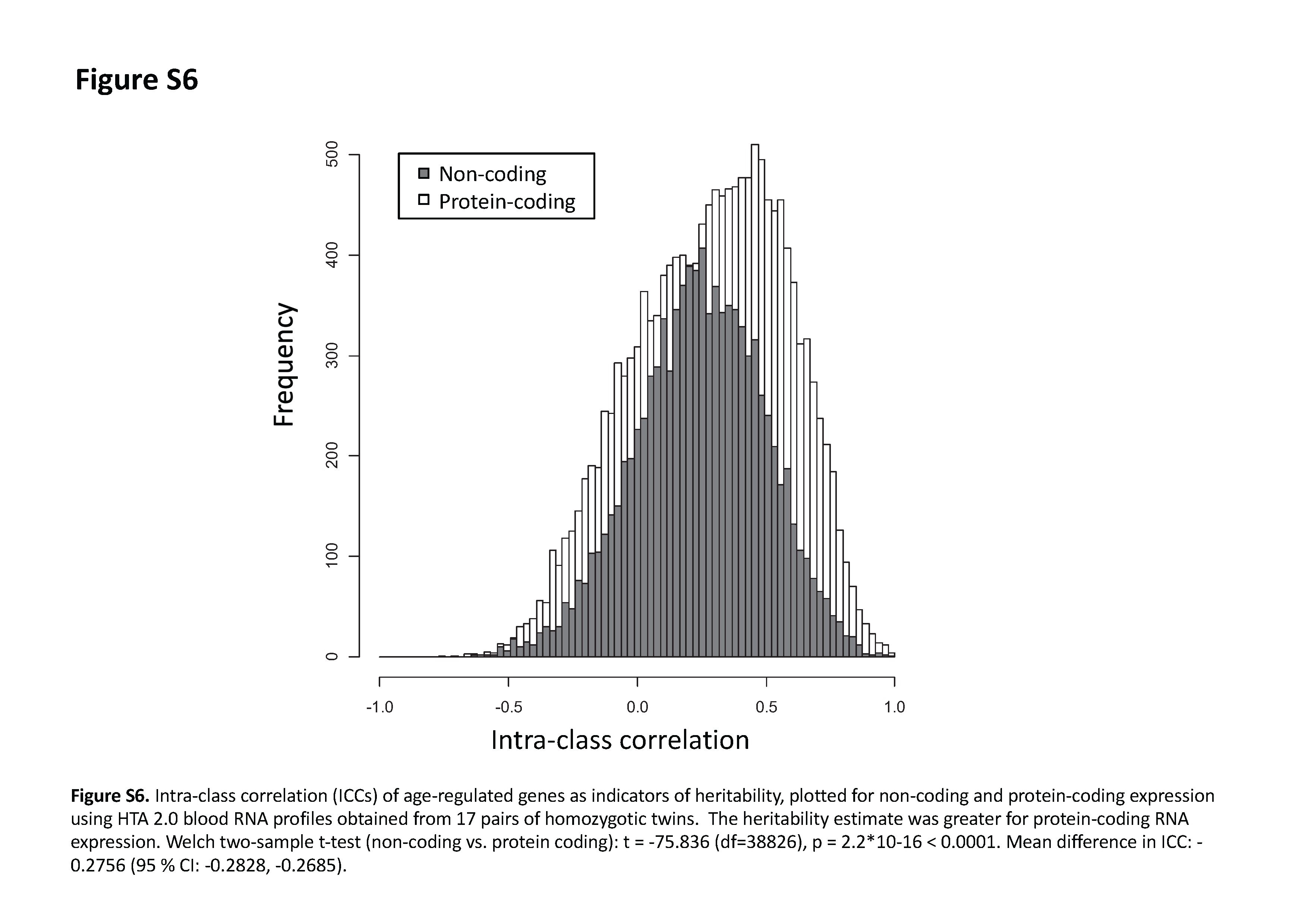

Supplement: Supplementary file 6 [file ACEL-18-e12970-s006.tiff]

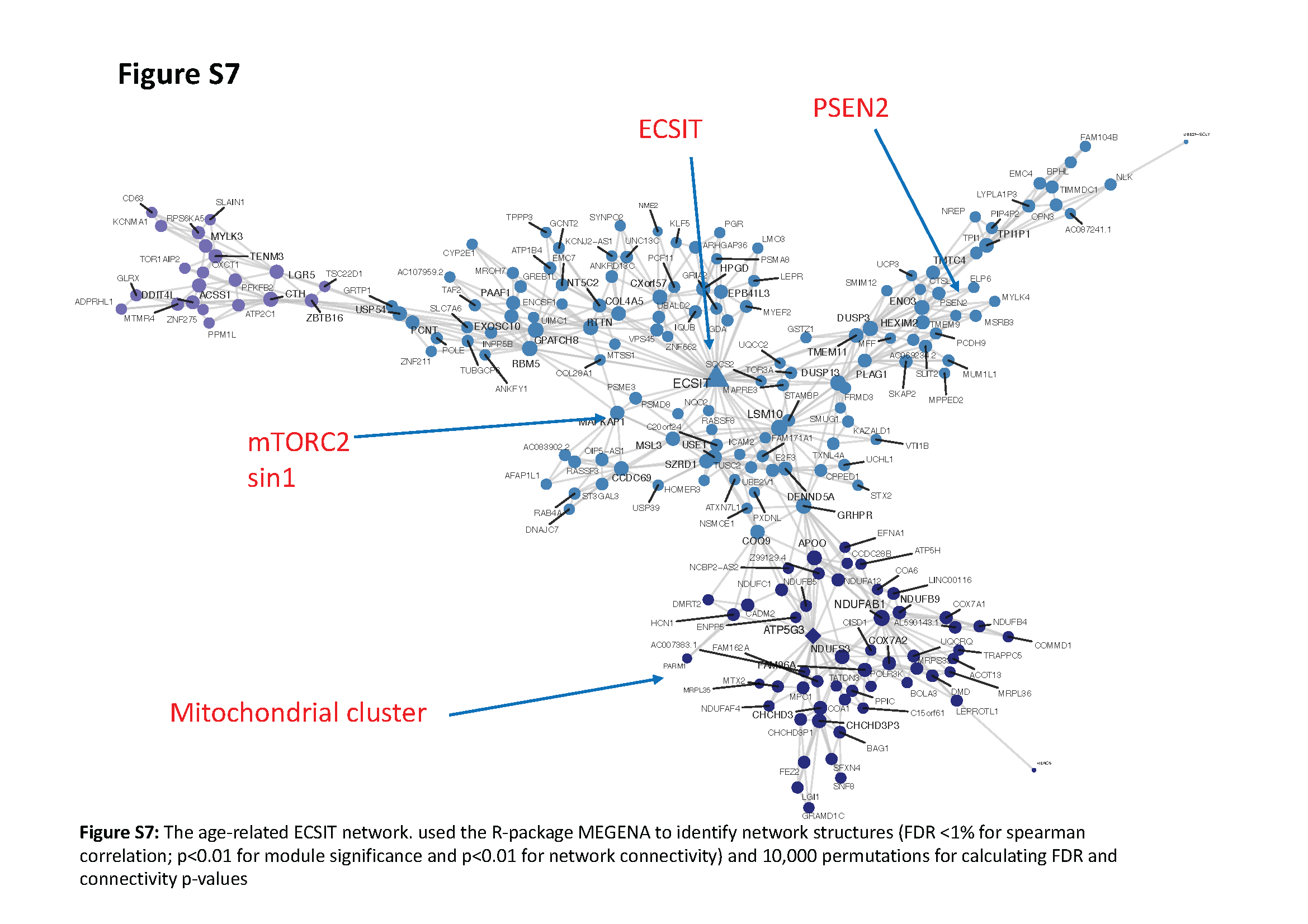

Supplement: Supplementary file 7 [file ACEL-18-e12970-s007.tiff]

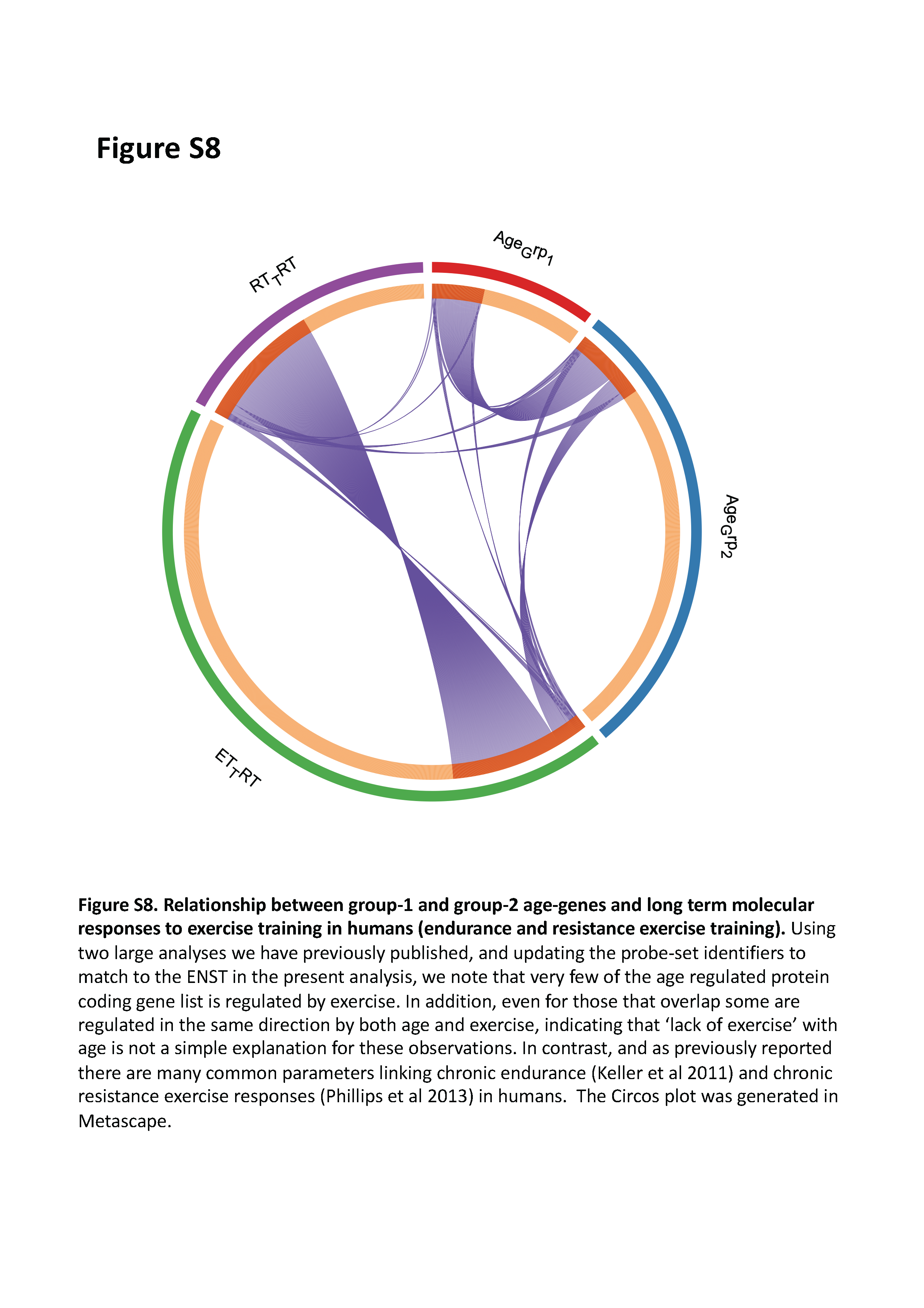

Supplement: Supplementary file 8 [file ACEL-18-e12970-s008.tiff]

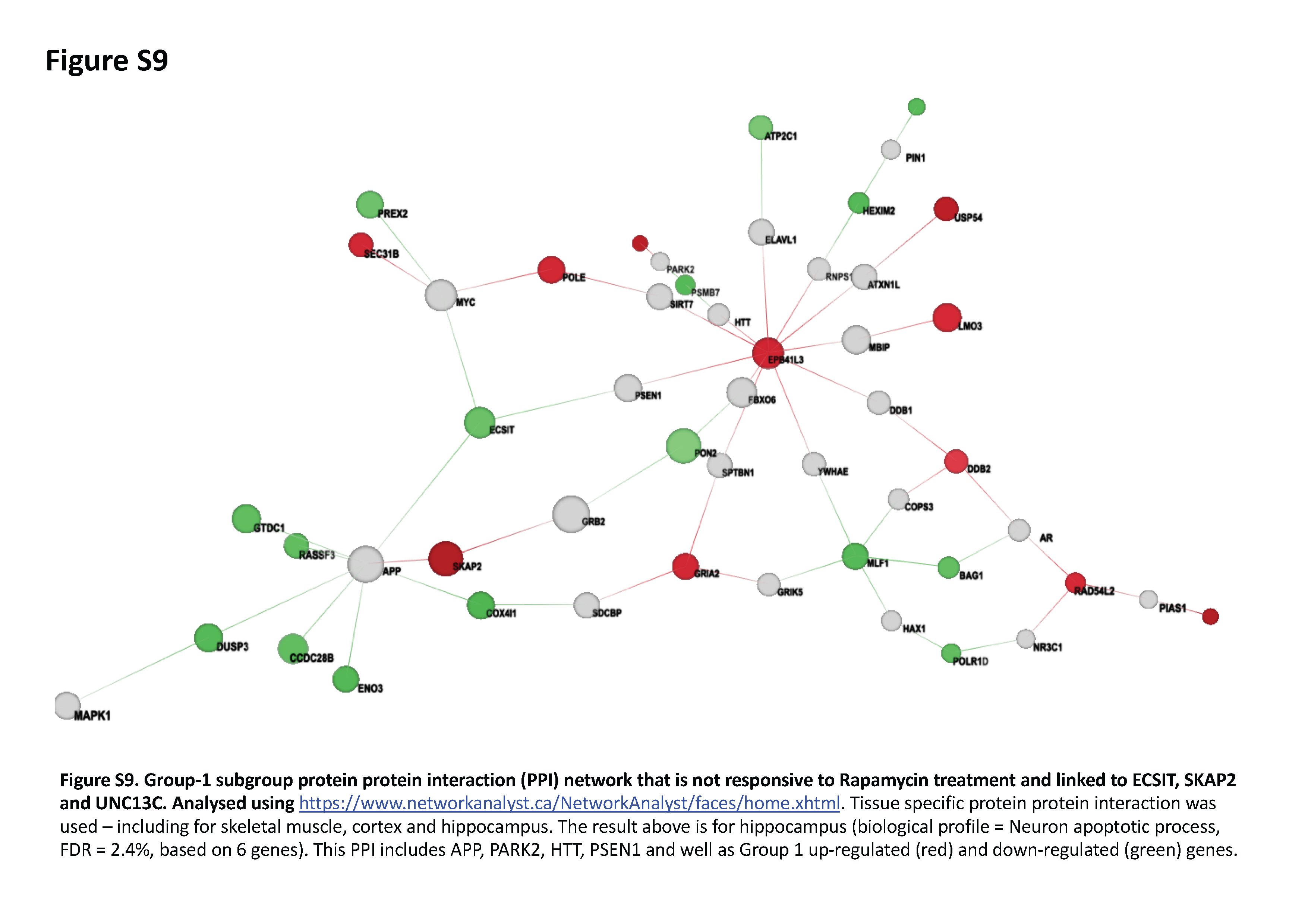

Supplement: Supplementary file 9 [file ACEL-18-e12970-s009.tiff]

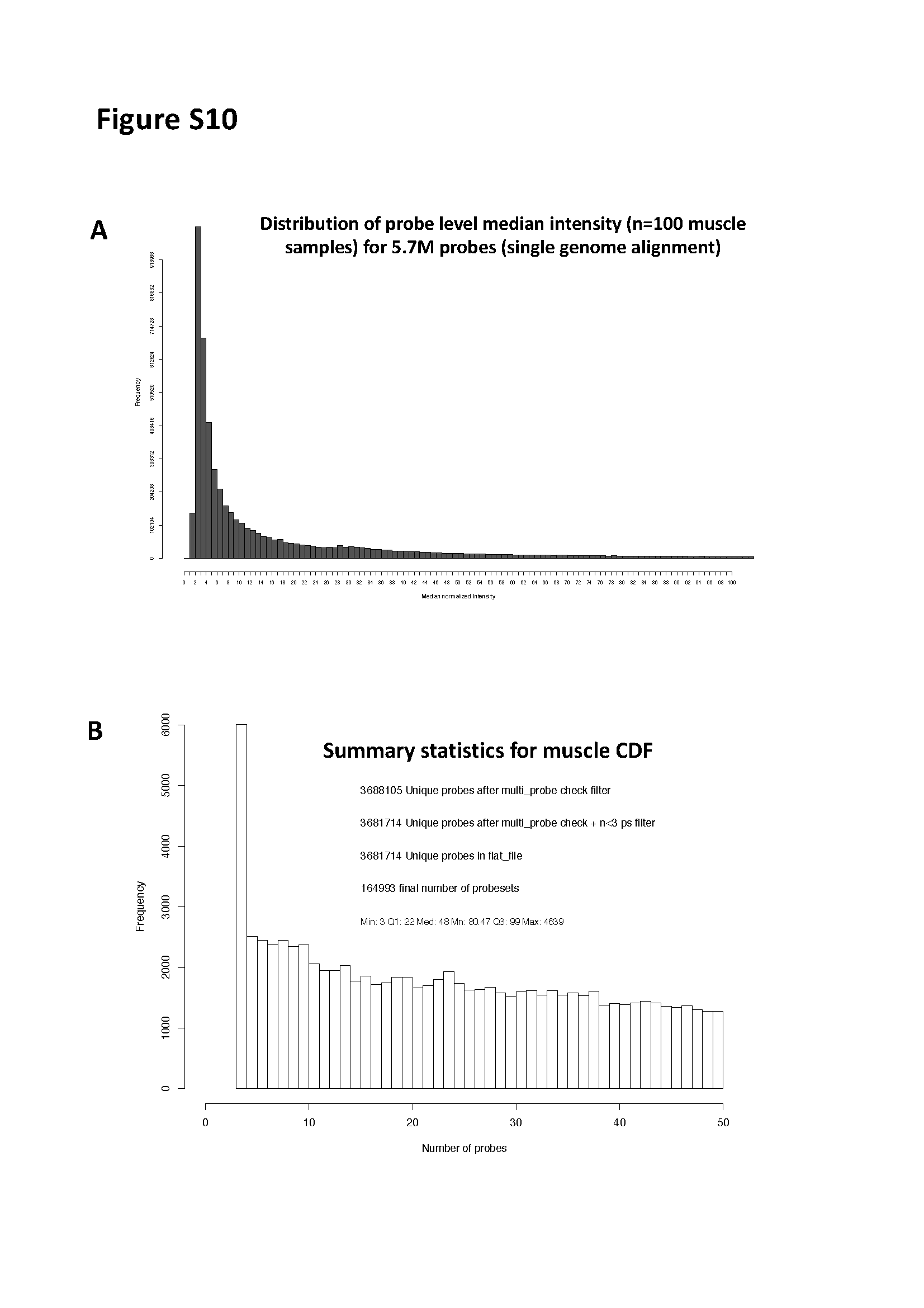

Supplement: Supplementary file 10 [file ACEL-18-e12970-s010.tiff]
