# Supplementary material for: Longevity‐related molecular pathways are subject to midlife “switch” in humans
Source: Aging Cell. 2019 Jun 6;18(4):e12970. doi: 10.1111/acel.12970 (PMC6612641; doi:10.1111/acel.12970)
Supplement: Supplementary file 11 [file ACEL-18-e12970-s011.docx]

**Online Experimental Procedures and Supplemental data and Tables**

All clinical studies complied with the 2008 Declaration of Helsinki and RNA profiling was approved by the relevant ethics committees stated in each clinical article, with all participants giving written, informed consent (Phillips et al. 2017; Gallagher et al. 2010; Phillips et al. 2013; AbouAssi et al. 2015; Slentz et al. 2016; Timmons et al. 2018). A subset of the large biobank (pre-intervention) has been utilized in the present analysis and the clinical characteristics can be found in Table S1 (Below).

There were four (A-D) main components to the analysis plan (Figure 1). **A)** Our recently developed RNA quantification method (Timmons et al. 2018) provides an enhanced signal for ensembl-based transcripts (ENST, [www.ensembl.org](http://www.ensembl.org)) ‘probe-sets’, was applied to probe-level Human Transcriptome Array 2.0. (HTA) data (Figure S1A and S1B). **B)** We curated a biobank of over 1,000 human skeletal muscle transcriptomic profiles from individuals physiologically phenotyped (Gallagher et al. 2010; Nakhuda et al. 2016; Hangelbroek et al. 2016; Slentz et al. 2005; Glynn et al. 2015; Slentz et al. 2016; Barberio et al. 2016; Cotie et al. 2014; Phillips et al. 2017; Josse et al. 2011; Tieland et al. 2012; AbouAssi et al. 2015; Sood et al. 2016; Phillips et al. 2013); including >500 splicing enabled Array profiles. Older-generation transcriptomic data (GSE47969, GSE73142, GSE18732 and GSE47874) was re-processed to match *transcript* identifiers from the HTA analysis (Timmons et al. 2018), resulting in 73,654 protein coding ENSTs common to all data-sets. **C)** Unlike tissue RNA-sequencing (RNA-seq), the HTA platform provides excellent quantification of tissue ncRNAs (Sood et al. 2016; Timmons et al. 2018). **D)** As 99% of ncRNAs remain to be functionally characterized extensively *in vitro*, *in silico* and network modeling strategies relied on protein-coding RNA to provide a functional context for the age-regulated lncRNAs.

All of the new HTA 2.0 array data has been deposited at GEO (GSE104235). The remainder of our existing array data is also available at GEO (GSE47969, GSE47881, GSE48278, GSE18732, and GSE73142). We also utilized two human brain data-sets on Exon arrays from GEO (GSE25219 and GSE46706), one neuronal cell-line data on HTA 2.0 (GSE21450) and one Human skin data-set (E-GEOD-18876) on Exon arrays. The muscle primary cell Rapamycin HTA 2.0 array data is also deposited at GEO (GSE104235). Standard quality control processes were performed for each data-set using NUSE plots and PCA. Less than 5% of our samples were lost due to gene-chip quality control and up to 30% array files within each GEO data set did not pass this process or were excluded as the patients were taking Statins and other chronic drug therapies. Additionally, several arrays failed a gender-check (X Chromosome specific gene expression versus reported gender in clinical data). These arrays should be removed from GEO to prevent potentially inaccurate meta-analysis.

**Human cell studies**

Human primary skeletal muscle cells were isolated from muscle biopsies from healthy adults as previously described (Crossland et al. 2016; Crossland et al. 2017). Myogenic cell enrichment used magnetic-activated cell sorting (MACS) and anti-CD56 microbeads (130-050-401; Miltenyi Biotec). Myogenic purity was assessed through measurement of fractional desmin positivity (Crossland et al. 2016; Crossland et al. 2017). Cells were washed in PBS and fixed in ice-cold 1:1 acetone/methanol, before being blocked in 5% (v/v) goat serum for 30 min at room temperature. Cells were subsequently incubated with rabbit anti-desmin monoclonal antibody (ab32362; Abcam) for 1h at room temperature, washed, and incubated with anti-rabbit TRITC-conjugated secondary antibody as previously detailed (Crossland et al. 2016; Crossland et al. 2017). Finally, cells were washed in PBS and mounted/DAPI-stained using Fluoroshield^TM^ mounting medium with DAPI. Myoblasts at passage 5-6 were cultured on Collagen Type I-coated 6-well dishes in Dulbecco’s Modified Eagle Medium/Nutrient Mixture F-12 (DMEM/F-12; Life Technologies) containing 20% (v/v) fetal bovine serum (FBS, Sigma-Aldrich), 1% (v/v) antibiotic-antimycotic (AbAm) solution and 4mM L-glutamine (Life Technologies). Cells were maintained at 37^o^C with 5% CO_2_. Once cells reached ~95% confluency, differentiation was induced by switching the medium to DMEM/F-12 containing 2% (v/v) horse serum (Sigma-Aldrich), 4mM L-glutamine and 1% (v/v) AbAm solution (Life Technologies). Six days following the initiation of differentiation, a media change was carried out, using differentiation media supplemented with long R3 IGF-1 (10 ng/ml; Sigma-Aldrich), with or without 100 nM rapamycin (Sigma-Aldrich). DMSO (final concentration 0.01% (v/v)) was used as a vehicle control for rapamycin. Samples were collected at 0h baseline and following 4h and 24h treatment.

Proteins were extracted from cells by repeatedly passing through gel-loading pipette tips. Samples were centrifuged at 13,000 g for 10 min at 4^o^C to remove cell debris. Protein samples (10 µg) were loaded onto Criterion XT 12% Bis-Tris gels (Bio-Rad) at 200V for 1h, then transferred to PVDF membrane for 1h at 100V. Next, membranes were blocked using 2.5% (w/v) BSA for 1h at room temperature and incubated with primary antibodies all diluted 1:2000 (phosphorylated mTOR Ser2448 (#2971) and phosphorylated 4E-BP1 Thr37/46 (#9459); all from Cell Signaling Technology) overnight at 4^o^C. The following day, membranes were washed 3x5 min with 1x TBS-Tween and incubated with HRP-conjugated anti-rabbit secondary antibody (New England) 1:2000 for 1h at room temperature. Bands were visualised by incubating with enhanced chemiluminescence detection reagent (Millipore) and exposing in a Chemidoc XRS system (Bio-Rad). Bands were normalized against Coomassie Brilliant Blue staining of the membrane.

Cells were harvested in TRIzol (Life Technologies) for total RNA isolation, or homogenisation buffer (50 mM Tris-HCl, pH 7.5, 1 mM EDTA, 1 mM EGTA, 10 mM ß-glycerophosphate, 50 mM NaF and complete protease inhibitor cocktail tablet (Roche, West Sussex, UK)) for protein extraction. RNA was suspended in 20 μl of RNase-free water and assessed using a NanoDrop (Thermo Scientific) and 2100 Bioanalyzer (Agilent) and frozen until used in the HTA 2.0 array protocol. Detailed methods describing the use of 18 hr of Paraquat treatment to increase ROS production in SHSY-5Y cells *in vitro* can be found in the authors original publication (Lenzken et al. 2011). We processed the cell line array data as per Figure 1A (See details below). Differential expression analysis was carried using limma packge in R (eBayes and lmFIT functions) with p-values adjusted using the fdr method (Smyth 2005) and sample sizes for each comparison can be found in the figure legends and results sections.

**Production of experiment-specific CDF ‘maps’ for analysis of HTA arrays**

Each transcriptional ‘unit’ of expression was defined by establishing, using the HTA 2.0 platform, the detectable probe signals for each tissue-type (muscle, brain and skin), and then assembling these GC content corrected ‘active’ probes (Figure S1A) into ensembl ENST ‘units’ (probe-sets) using a custom chip definition file (CDF). Older generation data (Exon and U133+2 arrays) were processed using a tissue-specific CDF (Exon arrays) or the relevant brainarray ENST CDF (Dai et al. 2005) but only transcript units found in the HTA 2.0 annotation were utilized down-stream. In contrast to the Illumina Human BodyMap and Genotype-Tissue Expression (GTEx, (Mele et al. 2015)) projects, which find limited lncRNA in individual types of tissue, the HTA 2.0 is particularly effective at detecting >10,000 lncRNA *per* type (Xu et al. 2017; Sood et al. 2016). Indeed, the idea that many lncRNAs are expressed in a highly cell-type specific manner appears to be as a consequence of RNA-seq pipelines not efficiently detecting lncRNA and perhaps reflecting the lack of linearity between sequencing depth and transcript signal for lower expressed genes (Sood et al. 2016). Other structural problems with the technology include extreme gene ontology representation bias exist and so can yield false-positive ontology enrichment in regulated gene lists (Timmons et al. 2015).

While the HTA 2.0 platform represents a very robust laboratory technology, informatics pipelines have not evolved to maximize its performance. The standard RNA transcript ‘map’ (CDF) for this array combines probe signals into the final probe-set irrespective of whether each 25-mer probe represents an active ‘signal’ in the experiment. Indeed, many investigators have even utilized a ‘gene’ level CDF however this does not correspond, in most cases, to a valid biological transcript (Figure 1A) but rather to a hybrid of all potential variants (Arner et al. 2016; Böhm et al. 2016; Xu et al. 2011; Dai et al. 2005) and this approach should be avoided. To create a sequence specificity verified CDF, the ~6.9 million probe sequences (obtained from Affymetrix website) were aligned to the latest genome build e.g. GRCh38_82p3 (e.g. see <http://brainarray.mbni.med.umich.edu> for a detailed description) using bowtie alignment tool (Langmead & Salzberg 2012). As the physical design of each probe is fixed (and incorporates the features of the reference genome used at the time of design) probes which map to more than one part of the genome are discarded.

In our case the ENSEMBL database was used to define each ‘transcript unit’ of interest (ENST) i.e. 1 ENST = 1 probe-set. However, other reference transcriptomes can also be utilized at this step. Aroma.affymetrix (Bengtsson et al. 2008) and other R Bioconductor packages (Gentleman et al. 2004) were used to assemble and summarize the data. This first step, however, only updates the *annotation* of the 6.9 million short 25mer ‘probes’ and critically, it does not confirm if, in particular experiment, each probe represents part of an *actively* *transcribed* RNA species. While the combination of 3’ bias profiling and median summarization (Giorgi et al. 2010) limited this problem with earlier array technologies, it is a significant issue when probes cover the entire genomic region that could give rise to a transcript and this has until now, been ignored. Thus, to produce a more accurate and experiment specific CDF (incorporating experiment specific corrected signal (Figure S1) for each **probe** was determined during pre-processing (Bengtsson et al. 2008), and probes representing only background (technical) signal identified (Bolstad et al. 2003). For each tissue type or experiment a large peak of low signal probes (e.g. <10 signal units) with a high co-efficient of variation (e.g. >25%) were removed (Figure S10, discarding >2million probes). There are ~50,000 probes on the HTA 2.0 array which have an extreme GC content (i.e. <20%, >80%) and these were removed as well (the adjustment model used to correct GC content is not effective at extreme GC values).

All remaining probes were retained as long the final ENST probe-set contained at least 3 probes (Figure S10). Importantly, the *discarded* probes had on average a signal ~7 fold *lower* than the other probes belonging to the same final probe-set they (would have) belonged to. The net effect of this probe-level filter is to substantially increases the average signal for the final probe-set (Figure 1A and Figure S1) – removing variance that should reflect technical sources. This 2-step process generates an ‘experiment specific’ map (i.e. CDF) and it improved the detection of differentially expressed genes >5-fold over a standard ‘gene-level’ CDF (n=44 paired analysis). In addition, within the retained but low signal probe-sets, using qPCR (Gallagher et al. 2010) we identified known low abundance mRNA transcripts in muscle (BDNF, CT values ~35) and low abundance receptors or enzymes known to be expressed and physiologically active in neurons/brain (e.g. TRPV1; 2.3 log2 signal units, transient receptor potential cation channel subfamily V member 1 and CERS1; 2.2 log2 signal units, ceramide synthase).

**Statistical analysis of ENST expression versus age**

Preliminary analysis using human blood and skin transcriptomes (Gheorghe et al. 2014; Gheorghe et al. 2016; Haustead et al. 2016) reported some non-linear RNA changes with age. However, these observations were based on samples that included disease, drug-treatment and evidence of RNA degradation (See Below), and none identified a central role for mTOR or other ‘positive control’ type findings. The HTA 2.0 array profiles from snap-frozen human skeletal muscle RNA, was processed using the muscle specific CDF (~164K ENST probe-sets incorporating 3.7M probes). For the older U133+2 platform, 73,654 probe-sets (ENST) were common to the ‘muscle-specific’ HTA 2.0 profile (predominately protein-coding). Probe-set values were log2 transformed for each of the data-sets, across the age-ranges defined in Figure 1, and the relationship between gene-expression and chronological age was examined for all protein-coding ENST. P-value was generated in R using ANOVA, with two clinical covariates (log_10_ of the HOMA2 S% values, (Wallace et al. 2004) defining insulin sensitivity, and aerobic capacity (VO_2_ max)). A meta-analysis of the 4 sets of age-RNA p-values across the common ENST protein-coding probe-sets (~20-50y, Figure 1) used the Stouffer method and MetaDE (Wang et al. 2012). For the first 3 decades of adulthood (n=333), only ENSTs (FDR <5% in all four conditions) were taken forward. Thereafter, univariate Spearman rank regression coefficients were examined for consistency in each of the cohorts, and only transcripts which demonstrated a *consistent* directional relationship between Age and RNA were retained (Supplemental data 1). The same analysis was repeated for the second chronological age range. For the ncRNA analysis there were a total of 238 HTA 2.0 profiles available and the same process, as described for protein-coding RNAs, could not be applied as one older data set lacked measures of aerobic capacity. Significant adjusted relationships noted in Cohort D (n=124) were examined in Cohorts B and F (Table S1 and Figure 1).

In order to study the significant muscle age-related transcripts in other human tissues, we utilized three publicly available Exon based (generation prior to the HTA 2.0 platform, which lacks many of the lncRNA) array data-sets (Jaffe et al. 2014; Haustead et al. 2016; Soreq et al. 2017). Analysis was carried out with experiment specific CDFs created for brain and skin (as above). We utilized three brain-regions common to both post mortem brain-bank data-sets, i.e. cerebellar cortex, frontal cortex and hippocampus. Unlike snap-frozen skeletal muscle biopsy samples, post mortem samples are subject to RNA degradation and following quality control analysis, 92% of the Kang samples (reference) were deemed usable. Unfortunately >20% of the Soreq *et al.* large older samples had indications of RNA degradation (Soreq et al. 2017), and a number of the clinical samples did not have the correct gender profile for the X chromosome transcript, XIST and the clinical phenotype data reported by Soreq *et al.* (Soreq et al. 2017) and thus were discarded. We did not use the available larger RNA-seq brain databases because they did not include lncRNAs and on closer inspection contained numerous samples with the wrong gender (again based on XIST profiles).

In each case the average correlation coefficient (CC) is utilized, across the three regions, as these smaller sample sizes are inherently less reliable for identifying CC values (Schönbrodt & Perugini 2013; Gobbi & Jurman 2015). Modeling a transcript specific profile of human brain, covering the fifth to ninth decades of life proved challenging; with the exon-array data-set from Hardy and colleagues (Trabzuni et al. 2011) being the only sufficiently sized brain-dataset for meaningful linear analysis (Schönbrodt & Perugini 2013). One-hundred and eighty-three samples from the same three brain regions as used above, appeared robust (cerebellum, hippocampus and frontal cortex; 49-91 yr, Supplemental data 4). For the Exon skin profiles from Haustead *et al.* (Haustead et al. 2016) >15% failed NUSE/PCA evaluation, while a further 15% involved older adults that were taking chronic medication for age-related diseases, leaving 59 samples that could be analyzed. In all cases the CEL file used in the present analysis are listed in the data-file, Supplemental data 2.

**Use of the CMap-L1000v1 chemical database of transcriptome responses**

We carried our CMap Analysis using a data-base of ~8000 perturbagens (CMap-L1000v1) to identify chemical compounds mediators that mimic or oppose the linear age protein-coding signature (<https://clue.io/>) (Subramanian et al. 2017). A rank order of similarity between the Grp2 age signature and *in vitro* signatures is calculated, and this score is adjusted by a heuristic that considers the magnitude of RNA change as well as the overall specificity (compared with the data-base as a whole) called the ‘connectivity map score’ (Kolmogorov-Smirnov nominal p-value vs the null-distribution, an FDR adjusted p-value) to yield an overall ‘enrichment score’ (ES) ranging from -100 to 100. There are three classes of perturbagen; small-molecule chemicals (including approved drugs), short-hairpin RNA (shRNA) knock-down and CRISPR based gene deletion. However from the original analysis it would appear that the small-molecule chemical profiles are by far the most stable and informative (Subramanian et al. 2017). The database (gene annotation, July 2016) includes direct measurement of RNA transcripts for each of 978 genes (and 80 control genes). The age-related transcript is either present in the list of 978 genes, or in an experimentally derived surrogate value called a ‘Landmark Gene’. ‘Landmark Gene’ is one of the 978 genes that during the method development strongly represented the behavior of another, unmeasured RNA (Subramanian et al. 2017). For each experiment, biological replicates are linearly transformed to have mean of zero and a standard deviation of 1 (moderated Z-score). Moderation is based on weighting the signal from each biological replicate for Spearman correlation within each ‘experiment’. In the original publication, approximately 75% of the time ‘class’ scores were cell-independent, with 13% being cell-type specific and had a validation of ~90% (using a 137compound validation set).

**Network analysis**

We used the R-package MEGENA (Song & Zhang 2015) to identify network structures (FDR <1% for spearman correlation; p<0.01 for module significance and p<0.01 for network connectivity) and 10,000 permutations for calculating FDR and connectivity p-values. Stability of individual sub-clusters of a network are assessed based on the impact of a data-split on compactness on a parent cluster versus the impact of randomly permuting or inserting nodes. Network data-plots were produced using Fruchterman-Reingold force directed plotting (Song & Zhang 2015) within MEGENA. While network analysis is routinely carried out using small data-sets there is evidence that this will not yield reliable results (COHAIN et al. 2017), much like for correlation analysis with sample sizes in the range of 20-30 (Schönbrodt & Perugini 2013; Gobbi & Jurman 2015). Thus, we applied network analysis to the largest single grouping possible of HTA 2.0 arrays (RNA extraction and chip analysis run as a batch) using all samples aged 69 years or younger. The input list was 853 ENST (one per gene) but did not include a few microRNAs that were significantly related to age, as technical validation of microRNA responses, as measured by the HTA 2.0 remains to be fully evaluated.

**Heritability analysis of twin samples (Figures 4B, Table S2 and Supplemental Figure S6)**

Blood samples from 17 male monozygotic twin pairs (mean age 34, range 32-37) participating in the FITFATTWIN study (Rottensteiner et al. 2014; Sood et al. 2016) (ancillary to the population based FinnTwin16 Cohort study) were profiled on the HTA 2.0. FITFATTWIN study included an interview and a medical examination at the laboratory as previously reported (Rottensteiner et al. 2014; Sood et al. 2016). The participants had no chronic disease affecting the ability to exercise, no acute disease and no drug or alcohol abuse and thus can be considered healthy. Some of the twin pairs were discordant for physical activity (Sood et al. 2016; Rottensteiner et al. 2014). All experimental procedures and study protocols were approved by the Ethical Review Board for Human Research of the Central Finland Health Care District (29 September 2011) and the study was conducted in accordance with the Declaration of Helsinki. All participants volunteered and provided written, informed consent. From the twin pairs we calculated intraclass correlations reflecting heritability *(37).* The intraclass correlation (*r_icc_*) was based on the one-way ANOVA fixed effects model and computed as:

$r_{ICC}=\frac{{MS}_{B}-{MS}_{W}}{{MS}_{B}+\left( n-1 \right){MS}_{w}}$,

where *MS*=mean square, *B*=between subjects, and *W*=within subjects, n=sample size.

An *r_icc_* = 0 corresponds to upper limit of heritability and estimates *h*^2^ = 0, while at *r_icc_* = 1 heritability is estimated to be h^2^ = 1. We compared non-coding versus protein coding expressions among both ageing-related (Figure 4B) and non-aging-related (Figure 4C) expressions using Welch t-test. Welch t-tests were computed, and figures constructed using the base package of R version 3.4.3 (2017) and intraclass correlations were computed using the psych package (version 1.7.3.21).

**Links to our earlier Knn non-linear based model of healthy tissue age**

In the present study, and as expected only 9 genes that were linearly related to age during the first 3 decades, were also part of a Knn based non-linear 150-gene ‘healthy muscle age’ model related to cognitive health and mortality (Sood et al. 2015). Over 50 of these 150 genes have been **independently** shown to be regulated/involved with the biochemistry/ molecular biology/genetics of aging and dementia - remarkably more than any other human age-signature (Yu et al. 2015; Kamboh et al. 2012; Miller et al. 2013; Bereczki et al. 2016; Huh et al. 2016; Xu et al. 2016; Stricker & Reiser 2014; Kim et al. 2014; Maurer et al. 2012; Aird et al. 2016; Yamanaka et al. 2015; Li, Hu, et al. 2017; Sidhu et al. 2016; Crespo et al. 2014; Tan et al. 2016; Lawrence et al. 2018; Zirkel et al. 2018; Li, Yu, et al. 2017; Liu et al. 2018; Park et al. 2012; Su et al. 2018; Kumar et al. 2017; Cui et al. 2017; Yang et al. 2016; Zhang et al. 2017; Sun et al. 2015; Gao et al. 2014; Szatmari et al. 2013; Zhao et al. 2018; Choi et al. 2013; Spang et al. 2014; Su et al. 2017; Bar-Peled et al. 2012; Xiao et al. 2018; Shahmoradi et al. 2015; Verdaguer et al. 2015; Zallo et al. 2018; Doens et al. 2017; Vjetrovic et al. 2014; Hu et al. 2017; Bigot et al. 2012; Murray & Cameron 2017; Khoshnan et al. 2017; Walker et al. 2017; Novais et al. 2018; Severini et al. 2015; Shin et al. 2018; Bakkar et al. 2018; Cheng et al. 2018; Sinkevicius et al. 2018; Huang et al. 2018; XiYang et al. 2016; Stützer et al. 2013; Yaguchi et al. 2017; Boonen et al. 2016; Dumitriu et al. 2012; Clarke et al. 2017; Cervetto et al. 2016; Costa et al. 2014; Pasanen et al. 2018; Brito-Moreira et al. 2017). A few remaining genes are directly involved with neurogenesis (Sims et al. 2017; Novais et al. 2018; Chang et al. 2013) or other pathways indirectly linked to aging or neurobiology (mTORC-ubiquitin). A further 9 were already linked to ageing or dementia at the time we published and since (Glorioso et al. 2011; Reiser & Bernstein 2004; Taru et al. 2002; Taniguchi et al. 2009; Seshadri et al. 2010; Fonseca & Soriano 1995; Coraci et al. 2002; Micheau 2003; Giampietri et al. 2010; Kung et al. 2010; Huang et al. 2011; Wu et al. 2008; Varela et al. 2005; Jacinto et al. 2006; Patel et al. 2011; Romano et al. 2013; Matsuo et al. 1996). The present model has directly validated links to mTOR and other canonical aging processes, while we await independent analysis of many of the more novel genes identified in the present study.

**References**

AbouAssi H, Slentz C a, Mikus CR, Tanner CJ, Bateman L a, Willis LH, Shields a T, Piner LW, Elliott-Penry LE, Kraus E a, Huffman KM, Bales CW, Houmard J a & Kraus WE (2015) The Effects of Aerobic, Resistance and Combination Training on Insulin Sensitivity and secretion in Overweight Adults from STRRIDE AT/RT: A Randomized Trial. *J. Appl. Physiol.* 118, 1474–1482.

Aird KM, Iwasaki O, Kossenkov A V., Tanizawa H, Fatkhutdinov N, Bitler BG, Le L, Alicea G, Yang T-L, Johnson FB, Noma K & Zhang R (2016) HMGB2 orchestrates the chromatin landscape of senescence-associated secretory phenotype gene loci. *J. Cell Biol.* 215, 325–334.

Arner P, Sahlqvist AS, Sinha I, Xu H, Yao X, Waterworth D, Rajpal D, Loomis AK, Freudenberg JM, Johnson T, Thorell A, N????slund E, Ryden M & Dahlman I (2016) The epigenetic signature of systemic insulin resistance in obese women. *Diabetologia* 59, 2393–2405.

Bakkar N, Kovalik T, Lorenzini I, Spangler S, Lacoste A, Sponaugle K, Ferrante P, Argentinis E, Sattler R & Bowser R (2018) Artificial intelligence in neurodegenerative disease research: use of IBM Watson to identify additional RNA-binding proteins altered in amyotrophic lateral sclerosis. *Acta Neuropathol.* 135, 227–247. Available at: http://link.springer.com/10.1007/s00401-017-1785-8 [Accessed April 1, 2019].

Bar-Peled L, Schweitzer LD, Zoncu R & Sabatini DM (2012) Ragulator Is a GEF for the Rag GTPases that Signal Amino Acid Levels to mTORC1. *Cell* 150, 1196–1208. Available at: http://www.ncbi.nlm.nih.gov/pubmed/22980980 [Accessed January 24, 2018].

Barberio MD, Huffman KM, Giri M, Hoffman EP, Kraus WE & Hubal MJ (2016) Pyruvate Dehydrogenase Phosphatase Regulatory Gene Expression Correlates with Exercise Training Insulin Sensitivity Changes. *Med. Sci. Sports Exerc.* 48, 2387–2397.

Bengtsson H, Simpson K, Bullard J & Hansen K (2008) aroma.affymetrix: A generic framework in R for analyzing small to very large Affymetrix data sets in bounded memory. *Dep. Stat. Univ. California, Berkeley* 745, 1–9. Available at: http://www.stat.berkeley.edu/tech-reports/745.pdf.

Bereczki E, Francis PT, Howlett D, Pereira JB, Höglund K, Bogstedt A, Cedazo-Minguez A, Baek J-H, Hortobágyi T, Attems J, Ballard C & Aarsland D (2016) Synaptic proteins predict cognitive decline in Alzheimer’s disease and Lewy body dementia. *Alzheimer’s Dement.* 12, 1149–1158. Available at: https://linkinghub.elsevier.com/retrieve/pii/S1552526016302448 [Accessed April 1, 2019].

Bigot N, Beauchef G, Hervieu M, Oddos T, Demoor M, Boumediene K & Galéra P (2012) NF-κB Accumulation Associated with COL1A1 Trans activators Defects during Chronological Aging Represses Type I Collagen Expression through a –112/–61-bp Region of the COL1A1 Promoter in Human Skin Fibroblasts. *J. Invest. Dermatol.* 132, 2360–2367. Available at: http://www.ncbi.nlm.nih.gov/pubmed/22673730 [Accessed April 1, 2019].

Böhm A, Hoffmann C, Irmler M, Schneeweiss P, Schnauder G, Sailer C, Schmid V, Hudemann J, Machann J, Schick F, Beckers J, De Angelis MH, Staiger H, Fritsche A, Stefan N, Nieß AM, Häring HU & Weigert C (2016) TGF-β contributes to impaired exercise response by suppression of mitochondrial key regulators in skeletal muscle. *Diabetes* 65, 2849–2861.

Bolstad BM, Irizarry RA, Astrand M & Speed TP (2003) A comparison of normalization methods for high density oligonucleotide array data based on variance and bias. *Bioinformatics* 19, 185–193.

Boonen M, Staudt C, Gilis F, Oorschot V, Klumperman J & Jadot M (2016) Cathepsin D and its newly identified transport receptor SEZ6L2 can modulate neurite outgrowth. *J. Cell Sci.* 129, 557–568. Available at: http://www.ncbi.nlm.nih.gov/pubmed/26698217 [Accessed April 1, 2019].

Brito-Moreira J, Lourenco M V., Oliveira MM, Ribeiro FC, Ledo JH, Diniz LP, Vital JFS, Magdesian MH, Melo HM, Barros-Aragão F, de Souza JM, Alves-Leon S V., Gomes FCA, Clarke JR, Figueiredo CP, De Felice FG & Ferreira ST (2017) Interaction of amyloid-β (Aβ) oligomers with neurexin 2α and neuroligin 1 mediates synapse damage and memory loss in mice. *J. Biol. Chem.* 292, 7327–7337. Available at: http://www.ncbi.nlm.nih.gov/pubmed/28283575 [Accessed April 1, 2019].

Cervetto C, Vergani L, Passalacqua M, Ragazzoni M, Venturini A, Cecconi F, Berretta N, Mercuri N, D’Amelio M, Maura G, Mariottini P, Voci A, Marcoli M & Cervelli M (2016) Astrocyte-Dependent Vulnerability to Excitotoxicity in Spermine Oxidase-Overexpressing Mouse. *NeuroMolecular Med.* 18, 50–68. Available at: http://link.springer.com/10.1007/s12017-015-8377-3 [Accessed April 1, 2019].

Chang SL-Y, Chen S-Y, Huang H-H, Ko H-A, Liu P-T, Liu Y-C, Chen P-H & Liu F-C (2013) Ectopic Expression of Nolz-1 in Neural Progenitors Promotes Cell Cycle Exit/Premature Neuronal Differentiation Accompanying with Abnormal Apoptosis in the Developing Mouse Telencephalon C.-T. Chien, ed. *PLoS One* 8, e74975. Available at: http://www.ncbi.nlm.nih.gov/pubmed/24073229 [Accessed April 1, 2019].

Cheng Y, Liu P, Zheng Q, Gao G, Yuan J, Wang P, Huang J, Xie L, Lu X, Tong T, Chen J, Lu Z, Guan J & Wang G (2018) Mitochondrial Trafficking and Processing of Telomerase RNA TERC. *Cell Rep.* 24, 2589–2595. Available at: http://www.ncbi.nlm.nih.gov/pubmed/30184494 [Accessed April 1, 2019].

Choi HY, Liu Y, Tennert C, Sugiura Y, Karakatsani A, Kröger S, Johnson EB, Hammer RE, Lin W & Herz J (2013) APP interacts with LRP4 and agrin to coordinate the development of the neuromuscular junction in mice. *Elife* 2, e00220. Available at: http://www.ncbi.nlm.nih.gov/pubmed/23986861 [Accessed April 1, 2019].

Clarke T-K, Adams MJ, Davies G, Howard DM, Hall LS, Padmanabhan S, Murray AD, Smith BH, Campbell A, Hayward C, Porteous DJ, Deary IJ & McIntosh AM (2017) Genome-wide association study of alcohol consumption and genetic overlap with other health-related traits in UK Biobank (N=112 117). *Mol. Psychiatry* 22, 1376–1384. Available at: http://www.ncbi.nlm.nih.gov/pubmed/28937693 [Accessed March 6, 2019].

COHAIN A, DIVARANIYA AA, ZHU K, SCARPA JR, KASARSKIS A, ZHU J, CHANG R, DUDLEY JT & SCHADT EE (2017) EXPLORING THE REPRODUCIBILITY OF PROBABILISTIC CAUSAL MOLECULAR NETWORK MODELS. In *Biocomputing 2017*. pp.120–131.

Coraci IS, Husemann J, Berman JW, Hulette C, Dufour JH, Campanella GK, Luster AD, Silverstein SC & El-Khoury JB (2002) CD36, a class B scavenger receptor, is expressed on microglia in Alzheimer’s disease brains and can mediate production of reactive oxygen species in response to beta-amyloid fibrils. *Am. J. Pathol.* 160, 101–12. Available at: http://www.ncbi.nlm.nih.gov/pubmed/11786404 [Accessed April 1, 2019].

Costa E, Fernandes J, Ribeiro S, Sereno J, Garrido P, Rocha-Pereira P, Coimbra S, Catarino C, Belo L, Bronze-da-Rocha E, Vala H, Alves R, Reis F & Santos-Silva A (2014) Aging is Associated with Impaired Renal Function, INF-gamma Induced Inflammation and with Alterations in Iron Regulatory Proteins Gene Expression. *Aging Dis.* 5, 356–65. Available at: http://www.ncbi.nlm.nih.gov/pubmed/25489488 [Accessed April 1, 2019].

Cotie LM, Josse AR, Phillips SM & MacDonald MJ (2014) Endothelial function increases after a 16-week diet and exercise intervention in overweight and obese young women. *Biomed Res. Int.* 2014, 327395.

Crespo ÂC, Silva B, Marques L, Marcelino E, Maruta C, Costa S, Timóteo Â, Vilares A, Couto FS, Faustino P, Correia AP, Verdelho A, Porto G, Guerreiro M, Herrero A, Costa C, de Mendonça A, Costa L & Martins M (2014) Genetic and biochemical markers in patients with Alzheimer’s disease support a concerted systemic iron homeostasis dysregulation. *Neurobiol. Aging* 35, 777–785. Available at: https://linkinghub.elsevier.com/retrieve/pii/S0197458013005460 [Accessed April 1, 2019].

Crossland H, Atherton PJJ, Strömberg A, Gustafsson T, Timmons JAA, Stro mberg A, Gustafsson T, Timmons JAA, Stro mberg A, Gustafsson T, Timmons JAA, Strömberg A, Gustafsson T, Timmons JAA, omberg A, Gustafsson T, Timmons JAA, Stro mberg A, Gustafsson T, Timmons JAA, Stro mberg A, Gustafsson T, Timmons JAA, Strömberg A, Gustafsson T, Timmons JAA, omberg A, Gustafsson T & Timmons JAA (2016) A reverse genetics cell-based evaluation of genes linked to healthy human tissue age. *FASEB J.* 31, 1–14. Available at: http://europepmc.org/abstract/med/27698205.

Crossland H, Timmons JA & Atherton PJ (2017) A dynamic ribosomal biogenesis response is not required for IGF-1–mediated hypertrophy of human primary myotubes. *FASEB J.* 31, 5196–5207. Available at: http://www.ncbi.nlm.nih.gov/pubmed/28774889 [Accessed March 12, 2018].

Cui L, Cai Y, Cheng W, Liu G, Zhao J, Cao H, Tao H, Wang Y, Yin M, Liu T, Liu Y, Huang P, Liu Z, Li K & Zhao B (2017) A Novel, Multi-Target Natural Drug Candidate, Matrine, Improves Cognitive Deficits in Alzheimer’s Disease Transgenic Mice by Inhibiting Aβ Aggregation and Blocking the RAGE/Aβ Axis. *Mol. Neurobiol.* 54, 1939–1952. Available at: http://www.ncbi.nlm.nih.gov/pubmed/26899576 [Accessed April 1, 2019].

Dai M, Wang P, Boyd AD, Kostov G, Athey B, Jones EG, Bunney WE, Myers RM, Speed TP, Akil H, Watson SJ & Meng F (2005) Evolving gene/transcript definitions significantly alter the interpretation of GeneChip data. *Nucleic Acids Res* 33, e175.

Doens D, Valiente PA, Mfuh AM, X. T. Vo A, Tristan A, Carreño L, Quijada M, Nguyen VT, Perry G, Larionov O V., Lleonart R & Fernández PL (2017) Identification of Inhibitors of CD36-Amyloid Beta Binding as Potential Agents for Alzheimer’s Disease. *ACS Chem. Neurosci.* 8, 1232–1241. Available at: http://www.ncbi.nlm.nih.gov/pubmed/28150942 [Accessed April 1, 2019].

Dumitriu A, Latourelle JC, Hadzi TC, Pankratz N, Garza D, Miller JP, Vance JM, Foroud T, Beach TG & Myers RH (2012) Gene Expression Profiles in Parkinson Disease Prefrontal Cortex Implicate FOXO1 and Genes under Its Transcriptional Regulation G. Gibson, ed. *PLoS Genet.* 8, e1002794. Available at: http://www.ncbi.nlm.nih.gov/pubmed/22761592 [Accessed April 1, 2019].

Fonseca M & Soriano E (1995) Calretinin-immunoreactive neurons in the normal human temporal cortex and in Alzheimer’s disease. *Brain Res.* 691, 83–91. Available at: http://www.ncbi.nlm.nih.gov/pubmed/8590068 [Accessed April 1, 2019].

Gallagher IJ, Scheele C, Keller P, Nielsen AR, Remenyi J, Fischer CP, Roder K, Babraj J, Wahlestedt C, Hutvagner G, Pedersen BK & Timmons J a (2010) Integration of microRNA changes in vivo identifies novel molecular features of muscle insulin resistance in type 2 diabetes. *Genome Med.* 2, 9. Available at: http://europepmc.org/abstract/med/20353613 [Accessed July 13, 2014].

Gao X, Teng Y, Luo J, Huang L, Li M, Zhang Z, Ma Y-C & Ma L (2014) The survival motor neuron gene *smn-1* interacts with the U2AF large subunit gene *uaf-1* to regulate *Caenorhabditis elegans* lifespan and motor functions. *RNA Biol.* 11, 1148–1160. Available at: http://www.ncbi.nlm.nih.gov/pubmed/25483032 [Accessed April 1, 2019].

Gentleman RC, Carey VJ, Bates DM, Bolstad B, Dettling M, Dudoit S, Ellis B, Gautier L, Ge Y, Gentry J, Hornik K, Hothorn T, Huber W, Iacus S, Irizarry R, Leisch F, Li C, Maechler M, Rossini AJ, Sawitzki G, Smith C, Smyth G, Tierney L, Yang JY & Zhang J (2004) Bioconductor: open software development for computational biology and bioinformatics. *Genome Biol* 5, R80.

Gheorghe M, Schurmann C, Peters MJ & André G (2016) Blood RNA expression profiles undergo major changes during the seventh decade. *Oncotarget* 7, 71353–71361. Available at: http://www.ncbi.nlm.nih.gov/pubmed/27655681%0Ahttp://www.pubmedcentral.nih.gov/articlerender.fcgi?artid=PMC5342083.

Gheorghe M, Snoeck M, Emmerich M, Bäck T, Goeman JJ & Raz V (2014) Major aging-associated RNA expressions change at two distinct age-positions. *BMC Genomics* 15, 132.

Giampietri C, Petrungaro S, Coluccia P, Antonangeli F, Giannakakis K, Faraggiana T, Filippini A, Cossu G & Ziparo E (2010) c-Flip overexpression affects satellite cell proliferation and promotes skeletal muscle aging. *Cell Death Dis.* 1, e38–e38. Available at: http://www.ncbi.nlm.nih.gov/pubmed/21364645 [Accessed April 1, 2019].

Giorgi FM, Bolger AM, Lohse M & Usadel B (2010) Algorithm-driven Artifacts in median polish summarization of Microarray data. *BMC Bioinformatics* 11.

Glorioso C, Oh S, Douillard GG & Sibille E (2011) Brain molecular aging, promotion of neurological disease and modulation by sirtuin 5 longevity gene polymorphism. 41. Available at: http://www.ncbi.nlm.nih.gov/pubmed/20887790 [Accessed April 1, 2019].

Glynn EL, Piner LW, Huffman KM, Slentz CA, Elliot-Penry L, AbouAssi H, White PJ, Bain JR, Muehlbauer MJ, Ilkayeva OR, Stevens RD, Porter Starr KN, Bales CW, Volpi E, Brosnan MJ, Trimmer JK, Rolph TP, Newgard CB & Kraus WE (2015) Impact of combined resistance and aerobic exercise training on branched-chain amino acid turnover, glycine metabolism and insulin sensitivity in overweight humans. *Diabetologia* 58, 2324–2335.

Gobbi A & Jurman G (2015) A null model for pearson coexpression networks. *PLoS One* 10, e0128115. Available at: http://www.ncbi.nlm.nih.gov/pubmed/26030917 [Accessed May 17, 2017].

Hangelbroek RWJ, Fazelzadeh P, Tieland M, Boekschoten M V, Hooiveld GJE, van Duynhoven JPM, Timmons JA, Verdijk LB, de Groot LCP, van Loon LJC & Müller M (2016) Expression of protocadherin gamma in skeletal muscle tissue is associated with age and muscle weakness. *J. Cachexia. Sarcopenia Muscle* 7, 604–614. Available at: http://www.ncbi.nlm.nih.gov/pubmed/27239416 [Accessed July 18, 2016].

Haustead DJ, Stevenson A, Saxena V, Marriage F, Firth M, Silla R, Martin L, Adcroft KF, Rea S, Day PJ, Melton P, Wood FM & Fear MW (2016) Transcriptome analysis of human ageing in male skin shows mid-life period of variability and central role of NF-κB. *Sci. Rep.* 6. Available at: http://dx.doi.org/10.1038/srep26846.

Hu C, Zhang Y, Tang K, Luo Y, Liu Y & Chen W (2017) Downregulation of CITED2 contributes to TGFβ-mediated senescence of tendon-derived stem cells. *Cell Tissue Res.* 368, 93–104. Available at: http://www.ncbi.nlm.nih.gov/pubmed/28084522 [Accessed April 1, 2019].

Huang J, Liu P & Wang G (2018) Regulation of mitochondrion-associated cytosolic ribosomes by mammalian mitochondrial ribonuclease T2 (RNASET2). *J. Biol. Chem.* 293, 19633–19644. Available at: http://www.jbc.org/lookup/doi/10.1074/jbc.RA118.005433 [Accessed April 1, 2019].

Huang L, Wu X, Jiang H, Gao P, Kuang C, Wang K & Huang L (2011) Aging reduces susceptibility of vascular smooth muscle cells to H2O2-induced apoptosis through the down-regulation of Jagged1 expression in endothelial cells. *Int. J. Mol. Med.* 28, 207–13. Available at: http://www.ncbi.nlm.nih.gov/pubmed/21491077 [Accessed April 1, 2019].

Huh S, Baek S-J, Lee KHK-H, Whitcomb DJ, Jo J, Choi S-M, Kim DH, Park M-S, Lee KHK-H & Kim BC (2016) The reemergence of long-term potentiation in aged Alzheimer’s disease mouse model. *Sci. Rep.* 6, 29152.

Jacinto E, Facchinetti V, Liu D, Soto N, Wei S, Jung SY, Huang Q, Qin J & Su B (2006) SIN1/MIP1 Maintains rictor-mTOR Complex Integrity and Regulates Akt Phosphorylation and Substrate Specificity. *Cell* 127, 125–137. Available at: http://www.ncbi.nlm.nih.gov/pubmed/16962653 [Accessed January 24, 2018].

Jaffe AE, Shin J, Collado-Torres L, Leek JT, Tao R, Li C, Gao Y, Jia Y, Maher BJ, Hyde TM, Kleinman JE & Weinberger DR (2014) Developmental regulation of human cortex transcription and its clinical relevance at single base resolution. *Nat. Neurosci.* 18, 154–161.

Josse AR, Atkinson SA, Tarnopolsky MA & Phillips SM (2011) Increased Consumption of Dairy Foods and Protein during Diet- and Exercise-Induced Weight Loss Promotes Fat Mass Loss and Lean Mass Gain in Overweight and Obese Premenopausal Women. *J. Nutr.* 141, 1626–1634. Available at: http://jn.nutrition.org/cgi/doi/10.3945/jn.111.141028.

Kamboh MI, Demirci FY, Wang X, Minster RL, Carrasquillo MM, Pankratz VS, Younkin SG, Saykin a J, Jun G, Baldwin C, Logue MW, Buros J, Farrer L, Pericak-Vance M a, Haines JL, Sweet R a, Ganguli M, Feingold E, Dekosky ST, Lopez OL & Barmada MM (2012) Genome-wide association study of Alzheimer’s disease. *Transl. Psychiatry* 2, e117.

Khoshnan A, Sabbaugh A, Calamini B, Marinero SA, Dunn DE, Yoo JH, Ko J, Lo DC & Patterson PH (2017) IKKβ and mutant huntingtin interactions regulate the expression of IL-34: implications for microglial-mediated neurodegeneration in HD. *Hum. Mol. Genet.* 26, 4267–4277. Available at: http://www.ncbi.nlm.nih.gov/pubmed/28973132 [Accessed April 1, 2019].

Kim JH, Song P, Lim H, Lee J-HJH, Lee J-HJH, Park SA & Alzheimer’s Disease Neuroimaging Initiative (2014) Gene-Based Rare Allele Analysis Identified a Risk Gene of Alzheimer’s Disease T. Arendt, ed. *PLoS One* 9, e107983. Available at: http://www.ncbi.nlm.nih.gov/pubmed/25329708 [Accessed April 1, 2019].

Kumar V, Fleming T, Terjung S, Gorzelanny C, Gebhardt C, Agrawal R, Mall MA, Ranzinger J, Zeier M, Madhusudhan T, Ranjan S, Isermann B, Liesz A, Deshpande D, Häring H-U, Biswas SK, Reynolds PR, Hammes H-P, Peperkok R, Angel P, Herzig S & Nawroth PP (2017) Homeostatic nuclear RAGE–ATM interaction is essential for efficient DNA repair. *Nucleic Acids Res.* 45, 10595–10613. Available at: http://academic.oup.com/nar/article/45/18/10595/4079821 [Accessed April 1, 2019].

Kung AWC, Xiao S-M, Cherny S, Li GHY, Gao Y, Tso G, Lau KS, Luk KDK, Liu J, Cui B, Zhang M-J, Zhang Z, He J, Yue H, Xia W, Luo L, He S, Kiel DP, Karasik D, Hsu Y-H, Cupples LA, Demissie S, Styrkarsdottir U, Halldorsson B V., Sigurdsson G, Thorsteinsdottir U, Stefansson K, Richards JB, Zhai G, Soranzo N, Valdes A, Spector TD & Sham PC (2010) Association of JAG1 with Bone Mineral Density and Osteoporotic Fractures: A Genome-wide Association Study and Follow-up Replication Studies. *Am. J. Hum. Genet.* 86, 229–239. Available at: http://www.ncbi.nlm.nih.gov/pubmed/20096396 [Accessed April 1, 2019].

Langmead B & Salzberg SL (2012) Fast gapped-read alignment with Bowtie 2. *Nat. Methods* 9, 357–359.

Lawrence I, Bene M, Nacarelli T, Azar A, Cohen JZ, Torres C, Johannes G & Sell C (2018) Correlations between age, functional status, and the senescence-associated proteins HMGB2 and p16INK4a. *GeroScience* 40, 193–199. Available at: http://www.ncbi.nlm.nih.gov/pubmed/29651745 [Accessed April 1, 2019].

Lenzken SC, Romeo V, Zolezzi F, Cordero F, Lamorte G, Bonanno D, Biancolini D, Cozzolino M, Pesaresi MG, Maracchioni A, Sanges R, Achsel T, Carrì MT, Calogero RA & Barabino SML (2011) Mutant SOD1 and mitochondrial damage alter expression and splicing of genes controlling neuritogenesis in models of neurodegeneration. *Hum. Mutat.* 32, 168–182.

Li C, Yu L, Xue H, Yang Z, Yin Y, Zhang B, Chen M & Ma H (2017) Nuclear AMPK regulated CARM1 stabilization impacts autophagy in aged heart. *Biochem. Biophys. Res. Commun.* 486, 398–405. Available at: http://www.ncbi.nlm.nih.gov/pubmed/28315332 [Accessed April 1, 2019].

Li N, Hu P, Xu T, Chen H, Chen X, Hu J, Yang X, Shi L, Luo J & Xu J (2017) iTRAQ-based Proteomic Analysis of APPSw,Ind Mice Provides Insights into the Early Changes in Alzheimer’s Disease. *Curr. Alzheimer Res.* 14, 1109–1122. Available at: http://www.ncbi.nlm.nih.gov/pubmed/28730955 [Accessed April 1, 2019].

Liu Y, Wang T, Ji YJ, Johnson K, Liu H, Johnson K, Bailey S, Suk Y, Lu Y-N, Liu M & Wang J (2018) A C9orf72–CARM1 axis regulates lipid metabolism under glucose starvation-induced nutrient stress. *Genes Dev.* 32, 1380–1397. Available at: http://genesdev.cshlp.org/lookup/doi/10.1101/gad.315564.118 [Accessed April 1, 2019].

Matsuo A, Walker DG, Terai K & McGeer PL (1996) Expression of CD43 in human microglia and its downregulation in Alzheimer’s disease. *J. Neuroimmunol.* 71, 81–6. Available at: http://www.ncbi.nlm.nih.gov/pubmed/8982106 [Accessed April 1, 2019].

Maurer B, Rumpf T, Scharfe M, Stolfa DA, Schmitt ML, He W, Verdin E, Sippl W & Jung M (2012) Inhibitors of the NAD ^+^ -Dependent Protein Desuccinylase and Demalonylase Sirt5. *ACS Med. Chem. Lett.* 3, 1050–1053.

Mele M, Ferreira PG, Reverter F, DeLuca DS, Monlong J, Sammeth M, Young TR, Goldmann JM, Pervouchine DD, Sullivan TJ, Johnson R, Segre A V., Djebali S, Niarchou A, Consortium TG, Wright FA, Lappalainen T, Calvo M, Getz G, Dermitzakis ET, Ardlie KG & Guigo R (2015) The human transcriptome across tissues and individuals. *Science (80-. ).* 348, 660–665.

Micheau O (2003) Cellular FLICE-inhibitory protein: an attractive therapeutic target? *Expert Opin. Ther. Targets* 7, 559–573. Available at: http://www.ncbi.nlm.nih.gov/pubmed/12885274 [Accessed April 1, 2019].

Miller J a, Woltjer RL, Goodenbour JM, Horvath S & Geschwind DH (2013) Genes and pathways underlying regional and cell type changes in Alzheimer’s disease. *Genome Med.* 5, 48.

Murray ER & Cameron AJM (2017) Towards specific inhibition of mTORC2. *Aging (Albany. NY).* 9, 2461–2462. Available at: http://www.ncbi.nlm.nih.gov/pubmed/29232655 [Accessed January 24, 2018].

Nakhuda A, Josse AR, Gburcik V, Crossland H, Raymond F, Metairon S, Good L, Atherton PJ, Phillips SM & Timmons JA (2016) Biomarkers of browning of white adipose tissue and their regulation. *Am. J. Clin. Nutr.*, 557–565.

Novais A, Silva A, Ferreira AC, Falcão AM, Sousa N, Palha JA, Marques F & Sousa JC (2018) Adult Hippocampal Neurogenesis Modulation by the Membrane-Associated Progesterone Receptor Family Member Neudesin. *Front. Cell. Neurosci.* 12, 463. Available at: http://www.ncbi.nlm.nih.gov/pubmed/30534059 [Accessed April 1, 2019].

Park H, Kam T-I, Kim Y, Choi H, Gwon Y, Kim C, Koh J-Y & Jung Y-K (2012) Neuropathogenic role of adenylate kinase-1 in Aβ-mediated tau phosphorylation via AMPK and GSK3β. *Hum. Mol. Genet.* 21, 2725–2737. Available at: http://www.ncbi.nlm.nih.gov/pubmed/22419736 [Accessed April 1, 2019].

Pasanen P, Myllykangas L, Pöyhönen M, Kiviharju A, Siitonen M, Hardy J, Bras J, Paetau A, Tienari PJ, Guerreiro R & Verkkoniemi-Ahola A (2018) Genetics of dementia in a Finnish cohort. *Eur. J. Hum. Genet.* 26, 827–837. Available at: http://www.ncbi.nlm.nih.gov/pubmed/29476165 [Accessed March 6, 2019].

Patel A, Rees SD, Kelly MA, Bain SC, Barnett AH, Thalitaya D & Prasher VP (2011) Association of variants within APOE, SORL1, RUNX1, BACE1 and ALDH18A1 with dementia in Alzheimer’s disease in subjects with Down syndrome. *Neurosci. Lett.* 487, 144–148. Available at: http://www.ncbi.nlm.nih.gov/pubmed/20946940 [Accessed April 1, 2019].

Phillips B, Kelly B, Lilja M, Ponce-González J, Brogan R, Morris D, Gustafsson T, Kraus W, Atherton P, Vollaard N, Rooyackers O & Timmons J (2017) A practical and time-efficient high-intensity interval training programme modifies cardio-metabolic risk-factors in adults with risk-factors for Type II diabetes. *Front. Endocrinol. (Lausanne).*, 1–9.

Phillips BE, Williams JP, Gustafsson T, Bouchard C, Rankinen T, Knudsen S, Smith K, Timmons JA & Atherton PJ (2013) Molecular Networks of Human Muscle Adaptation to Exercise and Age G. Gibson, ed. *PLoS Genet.* 9, e1003389. Available at: http://www.plosgenetics.org/article/metrics/info%3Adoi%2F10.1371%2Fjournal.pgen.1003389 [Accessed March 21, 2013].

Reiser G & Bernstein H-G (2004) Altered expression of protein p42IP4/centaurin-alpha 1 in Alzheimer’s disease brains and possible interaction of p42IP4 with nucleolin. *Neuroreport* 15, 147–8.

Romano GH, Harari Y, Yehuda T, Podhorzer A, Rubinstein L, Shamir R, Gottlieb A, Silberberg Y, Pe’er D, Ruppin E, Sharan R & Kupiec M (2013) Environmental Stresses Disrupt Telomere Length Homeostasis J.-Q. Zhou, ed. *PLoS Genet.* 9, e1003721. Available at: http://www.ncbi.nlm.nih.gov/pubmed/24039592 [Accessed April 1, 2019].

Rottensteiner M, Pietiläinen KH, Kaprio J & Kujala UM (2014) Persistence or change in leisure-time physical activity habits and waist gain during early adulthood: A twin-study. *Obesity* 22, 2061–2070. Available at: http://www.ncbi.nlm.nih.gov/pubmed/24839266 [Accessed April 12, 2018].

Schönbrodt FD & Perugini M (2013) At what sample size do correlations stabilize? *J. Res. Pers.* 47, 609–612. Available at: https://www.sciencedirect.com/science/article/pii/S0092656613000858 [Accessed February 23, 2018].

Seshadri S, Fitzpatrick AL, Ikram MA, DeStefano AL, Gudnason V, Boada M, Bis JC, Smith A V, Carassquillo MM, Lambert JC, Harold D, Schrijvers EMC, Ramirez-Lorca R, Debette S, Longstreth WT, Janssens ACJW, Pankratz VS, Dartigues JF, Hollingworth P, Aspelund T, Hernandez I, Beiser A, Kuller LH, Koudstaal PJ, Dickson DW, Tzourio C, Abraham R, Antunez C, Du Y, Rotter JI, Aulchenko YS, Harris TB, Petersen RC, Berr C, Owen MJ, Lopez-Arrieta J, Varadarajan BN, Becker JT, Rivadeneira F, Nalls MA, Graff-Radford NR, Campion D, Auerbach S, Rice K, Hofman A, Jonsson P V, Schmidt H, Lathrop M, Mosley TH, Au R, Psaty BM, Uitterlinden AG, Farrer LA, Lumley T, Ruiz A, Williams J, Amouyel P, Younkin SG, Wolf PA, Launer LJ, Lopez OL, van Duijn CM, Breteler MMB, CHARGE Consortium, GERAD1 Consortium & EADI1 Consortium (2010) Genome-wide Analysis of Genetic Loci Associated With Alzheimer Disease. *JAMA* 303, 1832. Available at: http://www.ncbi.nlm.nih.gov/pubmed/20460622 [Accessed April 1, 2019].

Severini C, Lattanzi R, Maftei D, Marconi V, Ciotti MT, Petrocchi Passeri P, Florenzano F, Del Duca E, Caioli S, Zona C, Balboni G, Salvadori S, Nisticò R & Negri L (2015) Bv8/prokineticin 2 is involved in Aβ-induced neurotoxicity. *Sci. Rep.* 5, 15301. Available at: http://www.nature.com/articles/srep15301 [Accessed April 1, 2019].

Shahmoradi A, Radyushkin K & Rossner MJ (2015) Enhanced memory consolidation in mice lacking the circadian modulators Sharp1 and -2 caused by elevated Igf2 signaling in the cortex. *Proc. Natl. Acad. Sci. U. S. A.* 112, E3582-9. Available at: http://www.pnas.org/lookup/doi/10.1073/pnas.1423989112 [Accessed April 1, 2019].

Shin Y, Kim Y, Kim H, Shin N, Kim T, Kwon T, Choi J & Chang J-S (2018) RASAL3 preferentially stimulates GTP hydrolysis of the Rho family small GTPase Rac2. *Biomed. Reports*. Available at: http://www.spandidos-publications.com/10.3892/br.2018.1119 [Accessed April 1, 2019].

Sidhu VK, Huang BX, Desai A, Kevala K & Kim H-Y (2016) Role of DHA in aging-related changes in mouse brain synaptic plasma membrane proteome. *Neurobiol. Aging* 41, 73–85. Available at: http://www.ncbi.nlm.nih.gov/pubmed/27103520 [Accessed April 1, 2019].

Sims R, Van Der Lee SJ, Naj AC, Bellenguez C, Badarinarayan N, Jakobsdottir J, Kunkle BW, Boland A, Raybould R, Bis JC, Martin ER, Grenier-Boley B, Heilmann-Heimbach S, Chouraki V, Kuzma AB, Sleegers K, Vronskaya M, Ruiz A, Graham RR, Olaso R, Hoffmann P, Grove ML, Vardarajan BN, Hiltunen M, Nöthen MM, White CC, Hamilton-Nelson KL, Epelbaum J, Maier W, Choi SH, Beecham GW, Dulary C, Herms S, Smith A V., Funk CC, Derbois C, Forstner AJ, Ahmad S, Li H, Bacq D, Harold D, Satizabal CL, Valladares O, Squassina A, Thomas R, Brody JA, Qu L, Sánchez-Juan P, Morgan T, Wolters FJ, Zhao Y, Garcia FS, Denning N, Fornage M, Malamon J, Naranjo MCD, Majounie E, Mosley TH, Dombroski B, Wallon D, Lupton MK, Dupuis J, Whitehead P, Fratiglioni L, Medway C, Jian X, Mukherjee S, Keller L, Brown K, Lin H, Cantwell LB, Panza F, McGuinness B, Moreno-Grau S, Burgess JD, Solfrizzi V, Proitsi P, Adams HH, Allen M, Seripa D, Pastor P, Cupples LA, Price ND, Hannequin D, Frank-García A, Levy D, Chakrabarty P, Caffarra P, Giegling I, Beiser AS, Giedraitis V, Hampel H, Garcia ME, Wang X, Lannfelt L, Mecocci P, Eiriksdottir G, Crane PK, Pasquier F, Boccardi V, Henández I, Barber RC, Scherer M, Tarraga L, Adams PM, Leber M, Chen Y, Albert MS, Riedel-Heller S, Emilsson V, Beekly D, Braae A, Schmidt R, Blacker D, Masullo C, Schmidt H, Doody RS, Spalletta G, Jr WTL, Fairchild TJ, Bossù P, Lopez OL, Frosch MP, Sacchinelli E, Ghetti B, Yang Q, Huebinger RM, Jessen F, Li S, Kamboh MI, Morris J, Sotolongo-Grau O, Katz MJ, Corcoran C, Dunstan M, Braddel A, Thomas C, Meggy A, Marshall R, Gerrish A, Chapman J, Aguilar M, Taylor S, Hill M, Fairén MD, Hodges A, Vellas B, Soininen H, Kloszewska I, Daniilidou M, Uphill J, Patel Y, Hughes JT, Lord J, Turton J, Hartmann AM, Cecchetti R, Fenoglio C, Serpente M, Arcaro M, Caltagirone C, Orfei MD, Ciaramella A, Pichler S, Mayhaus M, Gu W, Lleó A, Fortea J, Blesa R, Barber IS, Brookes K, Cupidi C, Maletta RG, Carrell D, Sorbi S, Moebus S, Urbano M, Pilotto A, Kornhuber J, Bosco P, Todd S, Craig D, Johnston J, Gill M, Lawlor B, Lynch A, Fox NC, Hardy J, Albin RL, Apostolova LG, Arnold SE, Asthana S, Atwood CS, Baldwin CT, Barnes LL, Barral S, Beach TG, Becker JT, Bigio EH, Bird TD, Boeve BF, Bowen JD, Boxer A, Burke JR, Burns JM, Buxbaum JD, Cairns NJ, Cao C, Carlson CS, Carlsson CM, Carney RM, Carrasquillo MM, Carroll SL, Diaz CC, Chui HC, Clark DG, Cribbs DH, Crocco EA, Decarli C, Dick M, Duara R, Evans DA, Faber KM, Fallon KB, Fardo DW, Farlow MR, Ferris S, Foroud TM, Galasko DR, Gearing M, Geschwind DH, Gilbert JR, Graff-Radford NR, Green RC, Growdon JH, Hamilton RL, Harrell LE, Honig LS, Huentelman MJ, Hulette CM, Hyman BT, Jarvik GP, Abner E, Jin LW, Jun G, Karydas A, Kaye JA, Kim R, Kowall NW, Kramer JH, Laferla FM, Lah JJ, Leverenz JB, Levey AI, Li G, Lieberman AP, Lunetta KL, Lyketsos CG, Marson DC, Martiniuk F, Mash DC, Masliah E, McCormick WC, McCurry SM, McDavid AN, McKee AC, Mesulam M, Miller BL, Miller CA, Miller JW, Morris JC, Murrell JR, Myers AJ, O’Bryant S, Olichney JM, Pankratz VS, Parisi JE, Paulson HL, Perry W, Peskind E, Pierce A, Poon WW, Potter H, Quinn JF, Raj A, Raskind M, Reisberg B, Reitz C, Ringman JM, Roberson ED, Rogaeva E, Rosen HJ, Rosenberg RN, Sager MA, Saykin AJ, Schneider JA, Schneider LS, Seeley WW, Smith AG, Sonnen JA, Spina S, Stern RA, Swerdlow RH, Tanzi RE, Thornton-Wells TA, Trojanowski JQ, Troncoso JC, Van Deerlin VM, Van Eldik LJ, Vinters H V., Vonsattel JP, Weintraub S, Welsh-Bohmer KA, Wilhelmsen KC, Williamson J, Wingo TS, Woltjer RL, Wright CB, Yu CE, Yu L, Garzia F, Golamaully F, Septier G, Engelborghs S, Vandenberghe R, De Deyn PP, Fernadez CM, Benito YA, Thonberg H, Forsell C, Lilius L, Kinhult-Stählbom A, Kilander L, Brundin R, Concari L, Helisalmi S, Koivisto AM, Haapasalo A, Dermecourt V, Fievet N, Hanon O, Dufouil C, Brice A, Ritchie K, Dubois B, Himali JJ, Keene CD, Tschanz J, Fitzpatrick AL, Kukull WA, Norton M, Aspelund T, Larson EB, Munger R, Rotter JI, Lipton RB, Bullido MJ, Hofman A, Montine TJ, Coto E, Boerwinkle E, Petersen RC, Alvarez V, Rivadeneira F, Reiman EM, Gallo M, O’Donnell CJ, Reisch JS, Bruni AC, Royall DR, Dichgans M, Sano M, Galimberti D, St George-Hyslop P, Scarpini E, Tsuang DW, Mancuso M, Bonuccelli U, Winslow AR, Daniele A, Wu CK, Peters O, Nacmias B, Riemenschneider M, Heun R, Brayne C, Rubinsztein DC, Bras J, Guerreiro R, Al-Chalabi A, Shaw CE, Collinge J, Mann D, Tsolaki M, Clarimón J, Sussams R, Lovestone S, O’Donovan MC, Owen MJ, Behrens TW, Mead S, Goate AM, Uitterlinden AG, Holmes C, Cruchaga C, Ingelsson M, Bennett DA, Powell J, Golde TE, Graff C, De Jager PL, Morgan K, Ertekin-Taner N, Combarros O, Psaty BM, Passmore P, Younkin SG, Berr C, Gudnason V, Rujescu D, Dickson DW, Dartigues JF, Destefano AL, Ortega-Cubero S, Hakonarson H, Campion D, Boada M, Kauwe JK, Farrer LA, Van Broeckhoven C, Ikram MA, Jones L, Haines JL, Tzourio C, Launer LJ, Escott-Price V, Mayeux R, Deleuze JF, Amin N, Holmans PA, Pericak-Vance MA, Amouyel P, Van Duijn CM, Ramirez A, Wang LS, Lambert JC, Seshadri S, Williams J & Schellenberg GD (2017) *Rare coding variants in PLCG2, ABI3, and TREM2 implicate microglial-mediated innate immunity in Alzheimer’s disease*,

Sinkevicius KW, Morrison TR, Kulkarni P, Caffrey Cagliostro MK, Iriah S, Malmberg S, Sabrick J, Honeycutt JA, Askew KL, Trivedi M & Ferris CF (2018) *RNaseT2* knockout rats exhibit hippocampal neuropathology and deficits in memory. *Dis. Model. Mech.* 11, dmm032631. Available at: http://www.ncbi.nlm.nih.gov/pubmed/29752287 [Accessed April 1, 2019].

Slentz CA, Aiken LB, Houmard JA, Bales CW, Johnson JL, Tanner CJ, Duscha BD & Kraus WE (2005) Inactivity, exercise, and visceral fat. STRRIDE: a randomized, controlled study of exercise intensity and amount. *J Appl Physiol* 99, 1613–1618.

Slentz CA, Bateman LA, Willis LH, Granville EO, Piner LW, Samsa GP, Setji TL, Muehlbauer MJ, Huffman KM, Bales CW & Kraus WE (2016) Effects of exercise training alone vs a combined exercise and nutritional lifestyle intervention on glucose homeostasis in prediabetic individuals: a randomised controlled trial. *Diabetologia* 59, 2088–2098.

Smyth GK (2005) Limma: linear models for microarray data. In R. Gentleman, V. Carey, S. Dudoit, & W. H. R. Irizarry, eds. *Bioinformatics and Computational Biology Solutions using R and Bioconductor*. New York: Springer, pp.397―420.

Song WM & Zhang B (2015) Multiscale Embedded Gene Co-expression Network Analysis. *PLoS Comput. Biol.* 11, e1004574.

Sood S, Gallagher IJ, Lunnon K, Rullman E, Keohane A, Crossland H, Phillips BE, Cederholm T, Jensen T, van Loon LJC, Lannfelt L, Kraus WE, Atherton PJ, Howard R, Gustafsson T, Hodges A & Timmons JA (2015) A novel multi-tissue RNA diagnostic of healthy ageing relates to cognitive health status. *Genome Biol.* 16, 185. Available at: http://genomebiology.com/2015/16/1/185.

Sood S, Szkop KJ, Nakhuda A, Gallagher IJ, Murie C, Brogan RJ, Kaprio J, Kainulainen H, Atherton PJ, Kujala UM, Gustafsson T, Larsson O & Timmons JA (2016) iGEMS: An integrated model for identification of alternative exon usage events. *Nucleic Acids Res.* 44, 1–14. Available at: http://europepmc.org/abstract/med/27095197.

Soreq L, Rose J, Soreq E, Hardy J, Trabzuni D, Cookson MR, Smith C, Ryten M, Patani R & Ule J (2017) Major Shifts in Glial Regional Identity Are a Transcriptional Hallmark of Human Brain Aging. *Cell Rep.* 18, 557–570. Available at: http://dx.doi.org/10.1016/j.celrep.2016.12.011.

Spang N, Feldmann A, Huesmann H, Bekbulat F, Schmitt V, Hiebel C, Koziollek-Drechsler I, Clement AM, Moosmann B, Jung J, Behrends C, Dikic I, Kern A & Behl C (2014) RAB3GAP1 and RAB3GAP2 modulate basal and rapamycin-induced autophagy. *Autophagy* 10, 2297–2309. Available at: http://www.ncbi.nlm.nih.gov/pubmed/25495476 [Accessed March 6, 2019].

Stricker R & Reiser G (2014) Functions of the neuron-specific protein ADAP1 (centaurin-α1) in neuronal differentiation and neurodegenerative diseases, with an overview of structural and biochemical properties of ADAP1. *Biol. Chem.* 395, 1321–40.

Stützer I, Selevsek N, Esterházy D, Schmidt A, Aebersold R & Stoffel M (2013) Systematic Proteomic Analysis Identifies β-Site Amyloid Precursor Protein Cleaving Enzyme 2 and 1 (BACE2 and BACE1) Substrates in Pancreatic β-Cells. *J. Biol. Chem.* 288, 10536–10547. Available at: http://www.ncbi.nlm.nih.gov/pubmed/23430253 [Accessed April 1, 2019].

Su M-Y, Morris KL, Kim DJ, Fu Y, Lawrence R, Stjepanovic G, Zoncu R & Hurley JH (2017) Hybrid Structure of the RagA/C-Ragulator mTORC1 Activation Complex. *Mol. Cell* 68, 835-846.e3. Available at: http://www.ncbi.nlm.nih.gov/pubmed/29107538 [Accessed April 1, 2019].

Su Y, Wang P, Shen H, Sun Z, Xu C, Li G, Tong T & Chen J (2018) The protein kinase D1-mediated classical protein secretory pathway regulates the Ras oncogene-induced senescence response. *J. Cell Sci.* 131, jcs207217. Available at: http://www.ncbi.nlm.nih.gov/pubmed/29420297 [Accessed April 1, 2019].

Subramanian A, Narayan R, Corsello SM, Peck DD, Natoli TE, Lu X, Gould J, Davis JF, Tubelli AA, Asiedu JK, Lahr DL, Hirschman JE, Liu Z, Donahue M, Julian B, Khan M, Wadden D, Smith IC, Lam D, Liberzon A, Toder C, Bagul M, Orzechowski M, Enache OM, Piccioni FF, Johnson SA, Lyons NJ, Berger AH, Shamji AF, Brooks AN, Vrcic A, Flynn C, Rosains J, Takeda DY, Hu R, Davison D, Lamb J, Ardlie K, Hogstrom L, Greenside P, Gray NS, Clemons PA, Silver S, Wu XXXXX, Zhao W-N, Read-Button W, Wu XXXXX, Haggarty SJ, Ronco L V., Boehm JS, Schreiber SL, Doench JG, Bittker JA, Root DE, Wong B, Golub TR, Johnson SA, Lyons NJ, Berger AH, Shamji AF, Brooks AN, Vrcic A, Flynn C, Rosains J, Takeda DY, Hu R, Davison D, Lamb J, Ardlie K, Hogstrom L, Greenside P, Gray NS, Clemons PA, Silver S, Wu XXXXX, Zhao W-N, Read-Button W, Wu XXXXX, Haggarty SJ, Ronco L V., Boehm JS, Schreiber SL, Doench JG, Bittker JA, Root DE, Wong B & Golub TR (2017) A Next Generation Connectivity Map: L1000 Platform and the First 1,000,000 Profiles. *Cell* 171, 1437-1452.e17. Available at: http://linkinghub.elsevier.com/retrieve/pii/S0092867417313090.

Sun X, Li PP, Zhu S, Cohen R, Marque LO, Ross CA, Pulst SM, Chan HYE, Margolis RL & Rudnicki DD (2015) Nuclear retention of full-length HTT RNA is mediated by splicing factors MBNL1 and U2AF65. *Sci. Rep.* 5, 12521. Available at: http://www.nature.com/articles/srep12521 [Accessed April 1, 2019].

Szatmari EM, Oliveira AF, Sumner EJ & Yasuda R (2013) Centaurin-α1-Ras-Elk-1 signaling at mitochondria mediates β-amyloid-induced synaptic dysfunction. *J. Neurosci.* 33, 5367–74. Available at: http://www.jneurosci.org/cgi/doi/10.1523/JNEUROSCI.2641-12.2013 [Accessed April 1, 2019].

Tan Q, Yalamanchili HK, Park J, De Maio A, Lu H-C, Wan Y-W, White JJ, Bondar V V, Sayegh LS, Liu X, Gao Y, Sillitoe R V, Orr HT, Liu Z & Zoghbi HY (2016) Extensive cryptic splicing upon loss of RBM17 and TDP43 in neurodegeneration models. *Hum. Mol. Genet.* 25, 5083–5093. Available at: https://academic.oup.com/hmg/article-lookup/doi/10.1093/hmg/ddw337 [Accessed April 1, 2019].

Taniguchi N, Carames B, Ronfani L, Ulmer U, Komiya S, Bianchi ME & Lotz M (2009) Aging-related loss of the chromatin protein HMGB2 in articular cartilage is linked to reduced cellularity and osteoarthritis. *Proc. Natl. Acad. Sci.* 106, 1181–1186. Available at: http://www.pnas.org/cgi/doi/10.1073/pnas.0806062106 [Accessed April 1, 2019].

Taru H, Iijima K-I, Hase M, Kirino Y, Yagi Y & Suzuki T (2002) Interaction of Alzheimer’s beta -amyloid precursor family proteins with scaffold proteins of the JNK signaling cascade. *J. Biol. Chem.* 277, 20070–8. Available at: http://www.jbc.org/lookup/doi/10.1074/jbc.M108372200 [Accessed April 1, 2019].

Tieland M, Dirks ML, van der Zwaluw N, Verdijk LB, van de Rest O, de Groot LCPGM & van Loon LJC (2012) Protein Supplementation Increases Muscle Mass Gain During Prolonged Resistance-Type Exercise Training in Frail Elderly People: A Randomized, Double-Blind, Placebo-Controlled Trial. *J. Am. Med. Dir. Assoc.* 13, 713–719.

Timmons JA, Atherton PJ, Larsson O, Sood S, Blokhin IO, Brogan RJ, Volmar C-H, Josse AR, Slentz C, Wahlestedt C, Phillips SM, Phillips BE, Gallagher IJ & Kraus WE (2018) A coding and non-coding transcriptomic perspective on the genomics of human metabolic disease. *Nucleic Acids Res.* 46, 7772–7792. Available at: http://www.ncbi.nlm.nih.gov/pubmed/29986096 [Accessed March 2, 2019].

Timmons JA, Szkop KJ & Gallagher IJ (2015) Multiple sources of bias confound functional enrichment analysis of global -omics data. *Genome Biol.* 16, 186. Available at: http://genomebiology.com/2015/16/1/186.

Trabzuni D, Ryten M, Walker R, Smith C, Imran S, Ramasamy A, Weale ME & Hardy J (2011) Quality control parameters on a large dataset of regionally dissected human control brains for whole genome expression studies. *J. Neurochem.* 119, 275–82. Available at: http://www.ncbi.nlm.nih.gov/pubmed/21848658 [Accessed May 7, 2014].

Varela I, Cadiñanos J, Pendás AM, Gutiérrez-Fernández A, Folgueras AR, Sánchez LM, Zhou Z, Rodríguez FJ, Stewart CL, Vega JA, Tryggvason K, Freije JMP & López-Otín C (2005) Accelerated ageing in mice deficient in Zmpste24 protease is linked to p53 signalling activation. *Nature* 437, 564–568. Available at: http://www.ncbi.nlm.nih.gov/pubmed/16079796 [Accessed April 1, 2019].

Verdaguer E, Brox S, Petrov D, Olloquequi J, Romero R, de Lemos ML, Camins A & Auladell C (2015) Vulnerability of calbindin, calretinin and parvalbumin in a transgenic/knock-in APPswe/PS1dE9 mouse model of Alzheimer disease together with disruption of hippocampal neurogenesis. *Exp. Gerontol.* 69, 176–188. Available at: http://www.ncbi.nlm.nih.gov/pubmed/26099796 [Accessed April 1, 2019].

Vjetrovic J, Shankaranarayanan P, Mendoza-Parra MA & Gronemeyer H (2014) Senescence-secreted factors activate Myc and sensitize pretransformed cells to TRAIL-induced apoptosis. *Aging Cell* 13, 487–496. Available at: http://www.ncbi.nlm.nih.gov/pubmed/24589226 [Accessed April 1, 2019].

Walker DG, Tang TM & Lue L-F (2017) Studies on Colony Stimulating Factor Receptor-1 and Ligands Colony Stimulating Factor-1 and Interleukin-34 in Alzheimer’s Disease Brains and Human Microglia. *Front. Aging Neurosci.* 9, 244. Available at: http://www.ncbi.nlm.nih.gov/pubmed/28848420 [Accessed April 1, 2019].

Wallace TM, Levy JC, Matthews DR & Homa T (2004) Use and Abuse of HOMA Modeling. *Diabetes Care* 27, 1487–1495.

Wang X, Kang DD, Shen K, Song C, Lu S, Chang LC, Liao SG, Huo Z, Tang S, Ding Y, Kaminski N, Sibille E, Lin Y, Li J & Tseng GC (2012) An r package suite for microarray meta-analysis in quality control, differentially expressed gene analysis and pathway enrichment detection. *Bioinformatics* 28, 2534–2536.

Wu X, Zhou Q, Huang L, Sun A, Wang K, Zou Y & Ge J (2008) Ageing-exaggerated proliferation of vascular smooth muscle cells is related to attenuation of Jagged1 expression in endothelial cells. *Cardiovasc. Res.* 77, 800–808. Available at: http://www.ncbi.nlm.nih.gov/pubmed/18079106 [Accessed April 1, 2019].

Xiao F-H, Chen X-Q, Yu Q, Ye Y, Liu Y-W, Yan D, Yang L-Q, Chen G, Lin R, Yang L, Liao X, Zhang W, Zhang W, Tang NL-S, Wang X-F, Zhou J, Cai W-W, He Y-H & Kong Q-P (2018) Transcriptome evidence reveals enhanced autophagy-lysosomal function in centenarians. *Genome Res.* 28, 1601–1610. Available at: http://genome.cshlp.org/lookup/doi/10.1101/gr.220780.117 [Accessed April 1, 2019].

XiYang Y-B, Wang Y-C, Zhao Y, Ru J, Lu B-T, Zhang Y-N, Wang N-C, Hu W-Y, Liu J, Yang J-W, Wang Z-J, Hao C-G, Feng Z-T, Xiao Z-C, Dong W, Quan X-Z, Zhang L-F & Wang T-H (2016) Sodium Channel Voltage-Gated Beta 2 Plays a Vital Role in Brain Aging Associated with Synaptic Plasticity and Expression of COX5A and FGF-2. *Mol. Neurobiol.* 53, 955–967. Available at: http://www.ncbi.nlm.nih.gov/pubmed/25575679 [Accessed April 1, 2019].

Xu J, Bai J, Zhang X, Lv Y, Gong Y, Liu L, Zhao H, Yu F, Ping Y, Zhang G, Lan Y, Xiao Y & Li X (2017) A comprehensive overview of lncRNA annotation resources. *Brief. Bioinform.* 18, 236–249.

Xu J, de Winter F, Farrokhi C, Rockenstein E, Mante M, Adame A, Cook J, Jin X, Masliah E & Lee K-F (2016) Neuregulin 1 improves cognitive deficits and neuropathology in an Alzheimer’s disease model. *Sci. Rep.* 6, 31692.

Xu W, Seok J, Mindrinos MN, Schweitzer AC, Jiang H, Wilhelmy J, Clark T a, Kapur K, Xing Y, Faham M, Storey JD, Moldawer LL, Maier R V, Tompkins RG, Wong WH, Davis RW & Xiao W (2011) Human transcriptome array for high-throughput clinical studies. *Proc. Natl. Acad. Sci. U. S. A.* 108, 3707–12.

Yaguchi H, Yabe I, Takahashi H, Watanabe M, Nomura T, Kano T, Matsumoto M, Nakayama KI, Watanabe M & Hatakeyama S (2017) Sez6l2 regulates phosphorylation of ADD and neuritogenesis. *Biochem. Biophys. Res. Commun.* 494, 234–241. Available at: http://www.ncbi.nlm.nih.gov/pubmed/29032200 [Accessed April 1, 2019].

Yamanaka Y, Faghihi MA, Magistri M, Alvarez-Garcia O, Lotz M & Wahlestedt C (2015) Antisense RNA controls LRP1 Sense transcript expression through interaction with a chromatin-associated protein, HMGB2. *Cell Rep.* 11, 967–76.

Yang C, Li X, Mo Y, Liu S, Zhao L, Ma X, Fang Z, Chen J, Chen Y, Yu X, Fang S, Zhang Y, Xian S & Wang Q (2016) β-Asarone Mitigates Amyloidosis and Downregulates RAGE in a Transgenic Mouse Model of Alzheimer’s Disease. *Cell. Mol. Neurobiol.* 36, 121–130. Available at: http://www.ncbi.nlm.nih.gov/pubmed/26271288 [Accessed April 1, 2019].

Yu L, Chibnik LB, Srivastava GP, Pochet N, Yang J, Xu J, Kozubek J, Obholzer N, Leurgans SE, Schneider JA, Meissner A, De Jager PL & Bennett DA (2015) Association of Brain DNA Methylation in *SORL1* , *ABCA7* , *HLA-DRB5* , *SLC24A4* , and *BIN1* With Pathological Diagnosis of Alzheimer Disease. *JAMA Neurol.* 72, 15.

Zallo F, Gardenal E, Verkhratsky A & Rodríguez JJ (2018) Loss of calretinin and parvalbumin positive interneurones in the hippocampal CA1 of aged Alzheimer’s disease mice. *Neurosci. Lett.* 681, 19–25. Available at: https://linkinghub.elsevier.com/retrieve/pii/S0304394018303628 [Accessed April 1, 2019].

Zhang Y, Bharathi SS, Rardin MJ, Lu J, Maringer K V., Sims-Lucas S, Prochownik E V., Gibson BW & Goetzman ES (2017) Lysine desuccinylase SIRT5 binds to cardiolipin and regulates the electron transport chain. *J. Biol. Chem.* 292, 10239–10249. Available at: http://www.ncbi.nlm.nih.gov/pubmed/28458255 [Accessed April 1, 2019].

Zhao K, Shen C, Li L, Wu H, Xing G, Dong Z, Jing H, Chen W, Zhang H, Tan Z, Pan J, Xiong L, Wang H, Cui W, Sun X-D, Li S, Huang X, Xiong W-C & Mei L (2018) Sarcoglycan Alpha Mitigates Neuromuscular Junction Decline in Aged Mice by Stabilizing LRP4. *J. Neurosci.* 38, 8860–8873. Available at: http://www.ncbi.nlm.nih.gov/pubmed/30171091 [Accessed April 1, 2019].

Zirkel A, Nikolic M, Sofiadis K, Mallm J-P, Brackley CA, Gothe H, Drechsel O, Becker C, Altmüller J, Josipovic N, Georgomanolis T, Brant L, Franzen J, Koker M, Gusmao EG, Costa IG, Ullrich RT, Wagner W, Roukos V, Nürnberg P, Marenduzzo D, Rippe K & Papantonis A (2018) HMGB2 Loss upon Senescence Entry Disrupts Genomic Organization and Induces CTCF Clustering across Cell Types. *Mol. Cell* 70, 730-744.e6. Available at: http://www.ncbi.nlm.nih.gov/pubmed/29706538 [Accessed April 1, 2019].

**Table S1. Over-view of independent clinical cohorts**

|  | **Cohort A** | **Cohort B** | **Cohort C** | **Cohort D** | **Cohort E** | **Cohort F^*^** | **Human Brain** | **Human Skin** |
| --- | --- | --- | --- | --- | --- | --- | --- | --- |
| **Number of profiles** | 97 | 48 | 161 | 124 | 68 | 91 | 299 | 59 |
| **RNA Platform** | U133+2 | U133+2 | U133+2 | HTA 2.0 | HTA 2.0 | HTA 2.0 | Exon ST | Exon ST |
| **Transcript unit** | ENST | ENST | ENST | ENST | ENST | ENST | ENST | ENST |
| **AGE** | 56  (23-77) | 25  (18-55) | 53  (41-68) | 34.4  (18-51) | 60  (45-75) | 68  (65-86) | 55  (18-101) | 45  (20-65) |
| **VO_2_ max** | 26.2  (10.6-55.8) | 35.9  (19.6-57.0) | 25.7  (13.2-41.8) | 27.3  (13.2-46.9) | 25.0  (16.5-40.6) | - | - | - |
| **Insulin sensitivity (log)** | 1.9  (0.7-2.5) | 2.0  (1.6-2.6) | 2.0  (1.4-2.8) | 1.9  (1.5-2.5) | 2.0  (1.2-2.5) | 1.7  (1.3-2.2) | - | - |

**Table S1.** Details of the 6 cohorts of sedentary adults (A(Gallagher et al. 2010), B(Phillips et al. 2013), C(Slentz et al. 2005; Slentz et al. 2016), D(Phillips et al. 2017), E(Glynn et al. 2015) and F(Hangelbroek et al. 2016)), confirmed free of treated chronic disease (*with the exception of Cohort F) used to study the age transcriptome in flash-frozen human muscle samples. The HTA 2.0 gene-chip data was updated and re-processed as described in the methods and those ensembl transcript identifiers (sets of probes combined – “probe-sets”) were identified, if possible, on the U133+2 and Exon ST gene-chips. In the case of the Exon ST chip, the exon with the highest expression value from the ENST unit was utilized. Values are total numbers or median (range). VO_2_ max is a measure of aerobic capacity (which is ~50% inherited, ml.min^-1^.kg^-1^). Insulin sensitivity is percentile insulin sensitivity from the HOMA2-IR model (Wallace et al. 2004). The human brain region exon-level array profiles (hippocampus, cerebellar cortex and frontal cortex) originate from two brain biobank projects (GSE25219 and GSE46706 (Jaffe et al. 2014; Trabzuni et al. 2011)) and the skin data (E-GEOD-18876) originates from a study of (largely) healthy skin aging (Haustead et al. 2016). In all three cases significant numbers of CEL files deposited at GEO demonstrated technical issues.

**Table S2. Summary statistics for Intra-class correlation distributions for age-related RNA and all ENST**

|  |  | | | | | |
| --- | --- | --- | --- | --- | --- | --- |
|  | **Aging-related** | | |  | **ALL ENST*** | |
|  |  |  |  |  |  |  |
|  |  |  |  |  |  |  |
| **Statistic** | **Non-coding** |  | **Protein** |  | **Non-coding** | **Protein** |
|  |  |  |  |  |  |  |
|  |  |  |  |  |  |  |
| Location |  |  |  |  |  |  |
| Mean | 0.239 |  | 0.377 |  | 0.189 | 0.379 |
| Median | 0.258 |  | 0.412 |  | 0.193 | 0.403 |
|  |  |  |  |  |  |  |
| Dispersion |  |  |  |  |  |  |
| SD | 0.278 |  | 0.239 |  | 0.273 | 0.254 |
| IQR | 0.391 |  | 0.316 |  | 0.393 | 0.352 |
|  |  |  |  |  |  |  |
| Shape |  |  |  |  |  |  |
| Skew | -0.284 |  | -0.443 |  | -0.066 | -0.442 |
| Kurtosis | -0.483 |  | -0.089 |  | -0.426 | -0.062 |
|  |  |  |  |  |  |  |
| Percentiles |  |  |  |  |  |  |
| Min | -0.455 |  | -0.354 |  | -0.667 | -0.752 |
| 25 % | 0.041 |  | 0.221 |  | -0.005 | 0.214 |
| 50 % | 0.258 |  | 0.412 |  | 0.193 | 0.403 |
| 75 % | 0.432 |  | 0.537 |  | 0.388 | 0.566 |
| Max | 0.762 |  | 0.944 |  | 0.981 | 0.994 |
|  |  |  |  |  |  |  |

***excluding the subset of age-regulated ENSTs**

**Table S2.** Summary statistics for Intra-class correlation distributions for age-related RNA and all ENST in HTA 2.0 profiled whole blood RNA from identical twins (Sood et al. 2016; Rottensteiner et al. 2014). The HTA 2.0 gene-chip data was updated and re-processed as described in the methods and those ensembl transcript identifiers (sets of probes combined – “probe-sets”) were identified.
